# Single step eco-efficient mild chemical process for the total valorisation of rice husk: a focus on the inorganics as a cement additive†

**DOI:** 10.1039/d4ra05263c

**Published:** 2024-11-13

**Authors:** Eleonora Conterosito, Geo Paul, Valentina Toson, Valentina Gianotti, Marco Milanesio, Daniela Gastaldi, Enrico Boccaleri

**Affiliations:** a Dipartimento per lo Sviluppo Sostenibile e la Transizione Ecologica, Università del Piemonte Orientale A. Avogadro piazza S. Eusebio 5 13100 Vercelli Italy enrico.boccaleri@uniupo.it; b Dipartimento di Scienze ed Innovazione Tecnologica, Università del Piemonte Orientale A. Avogadro viale T. Michel 11 15121 Alessandria Italy; c Built – Buzzi Innovation Lab and Technology Via Restano 3 13100 Vercelli Italy

## Abstract

The rice husk biomass remaining from the industrial processing of rice constitutes approximately 25 wt% of the edible rice produced, and its disposal is challenging due to its high silica content. Here, we describe the optimization of a single step innovative chemical process for the conversion of rice husk-based biomass into useable products which tackles all fractions of the input biomass. The chemical process consists of a single step hydrothermal low temperature treatment of rice husk biomass leading to three easy-to-recover fractions. With appropriate chemical treatments, each of these fractions can serve specific applications effectively, overcoming the issues present in the original biomass. This paper will present the treatment method and the optimization of chemical conditions for ideal fractionation as well as include the characterization of the recovered materials. Additionally, the paper will explore the use of one of these materials—the inorganic precipitate fraction (P), which is rich in calcium silicate hydrate (C–S–H) phase—as an additive to promote C–S–H nucleation in cementitious materials. The process also yields a liquid fraction (S) rich in sugars and soluble inorganic species, and a fibrous fraction (HR) containing lignin and cellulose residues. All these components were characterized to assess their suitability for potential applications. A detailed study on the application of these materials in the fields of plant biology and polymer science will be presented in (a) subsequent publication(s). The three fractions were characterized by a multi-technique approach involving PXRD, XRF, TGA/DSC, Electron microscopy and NMR. The above chemical process can be extended to any straw and husk-based cereal crops (wheat or barley), broadening and strengthening the bio-based industries and improving the circularity of food-related byproducts.

## Abstract 2

Rice production generates 25 wt% of rice husk as a waste, whose valorisation is challenging due to its high silica content. A mild chemical process separates it in three fractions, and the inorganic one can improve cementitious materials.

## Graphical abstract

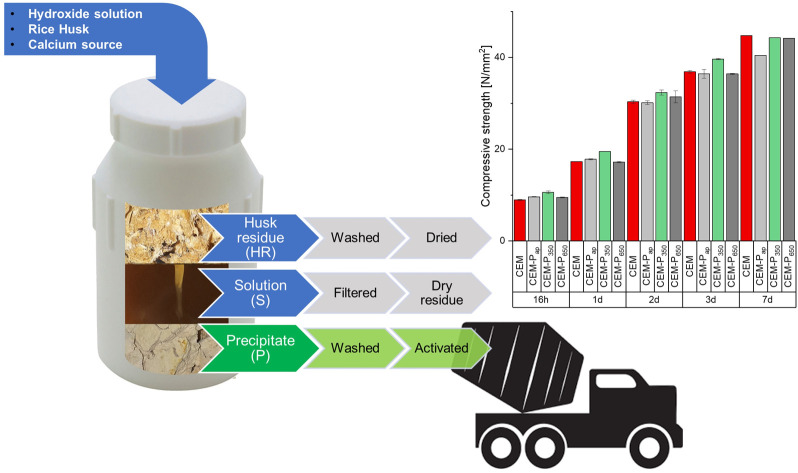

## Introduction

Rice and corn are considered the most important staple foods worldwide, meeting the primary dietary needs of nearly half the global population and serving as the main source of sustenance for a significant portion of people around the world.^1^ Rice husk and straw biomass are significant byproducts of rice production, constituting approximately 13% and 47% of the entire rice plant, respectively. Specifically for rice, one of these fractions is produced out of the field in industrial contexts. In fact, during the industrial processing of paddy rice, the inedible rice husk is removed from the grain, and this byproduct represents around 25% of the weight of the edible rice. This fraction is formally an industrial waste, and it is not currently fully used but can become an economically, environmentally, and socially sustainable source of bio-based materials with high potential. According to the United Nations, more than half of the world's 7.7 billion people eat rice as their staple food. The annual worldwide rice production was estimated to be about 800 million tons in 2017 and about 200 million tons of rice husk was generated during rice processing.^1,2^ Similarly, wheat and barley production was around 800 and 150 million tons, respectively. In several Mediterranean countries of Europe, rice has an important sociocultural and ecological importance. The total rice-growing area within the 28 European Union member countries is approximately 500 000 ha, with Italy, the leading European producer, accounting for a total of 250 000 ha. Straw from wheat and rice, and rice husk are widely available as residual fraction left in the fields or as industrial by-products. With current farming technologies and agricultural practices, both rice and wheat straw can be employed as feedstock. Despite their abundance, straw and husk from cereal crops have limited use and value and at present, only about 20% of rice straw is used for purposes such as production of ethanol, paper, fertilizers, and fodders, while almost no industrial use is made of rice husk. Most of the cereal straw and husk material is returned to soil, mixed in manure, burned directly in the fields or in specific plants for power and heat generation. Field burning of leftover cereal residues is forbidden in EU countries, although still practiced in several areas, since the combustion of these fractions can be harmful as, according to their composition, a spreading of fine silica particles can occur, with related concerns on human health and safety. It is also noteworthy that the presence of straw, and eventually husk, on the paddy fields can be highly noxious to the environment, since they are resistant to degradation, and the presence of water during flooding promotes their anaerobic degradation with production of methane, a powerful greenhouse gas with 25 times the effect of CO_2_. For this reason, paddy fields are listed in the IPCC reports on climate change as responsible for 7% of the global CH_4_ emissions in the atmosphere.^3,4^

Rice husk composition is very interesting because it represents one of the most available cellulose sources, based on its high cellulose fraction (∼33 wt%). Moreover, it contains hemicellulose (∼20 wt%), lignin (∼22 wt%) and silica (∼20 wt%).^5,6^ The directly soluble fraction is about 3 wt%. Starting from rice husk and straw composition, several methodologies are proposed in the literature for the recovery of the organic or the inorganic fraction.^7^ Acidic treatments with strong oxidizing acids, like HNO_3_, were evaluated to promote the digestion of organic materials.^8^ In other studies, cellulose nanocrystals were extracted from rice husk by combined treatments, involving alkali, bleaching and acid hydrolysis,^9^ or de-waxing by organic washing, delignification by NaClO_2_/CH_3_COOH and alkali with KOH.^10^ Reinforcement of polylactic acid was obtained adding cellulose fibers extracted from rice husk.^11^ The silica fraction can be recovered^7^ by calcination of rice husk and then used as source for acid resistant calcium silicate^12^ or as active silica source.^13^ Moreover, nanosilica can be obtained by extraction with acidic solutions (citric acid or acetic acid) and subsequent calcination at 650 °C.^14^

Multi-step procedures involving concurrent basic and acidic treatments of rice husk were also reported.^15^ Integral fractionation of rice husks using subcritical water extraction has also been reported.^16^ Such a process preserves the antioxidant and antibacterial activity of arabinoxylans compared to the alkaline process. The acidic treatment of rice husk to precipitate calcium silicate was also recently reported.^17^

In this work we exploit and optimize a mild and easy chemical process for the recovery of valuable fractions from rice husk biomass which operates in a single step and leads to three easy-to-recover products: an inorganic precipitate (P), a solution (S) and fibrous husk residues (HR).

The chemical process is a subcritical water extraction (SWE) under mild conditions using alkaline medium, with reaction conditions in line with green chemistry principles.^18^ It is performed in a single step (6^th^ and 8^th^ principle), it uses non-hazardous chemicals and does not produce hazardous wastes or fumes (3^rd^ principle) and each fraction obtained can be reused or valorized in some way (7^th^ principle).^13^

This extraction procedure requires an alkaline environment, a high concentration of Ca^2+^ ions and can be described by the following reactions:

1 SiO_2_(from the husk) + 2NaOH + H_2_O → Na_2_SiO_3(l)_ + 2H_2_O

2 Na_2_SiO_3(l)_ + CaCl_2(s)_ + H_2_O → CaSiO_3_ + 2Na^+^ + 2Cl^−^ + H_2_O

3 CaSiO_3_ + H_2_O → CaO·SiO_2_·*X*H_2_O (↓)

which represent respectively:

(1) Alkaline-catalysed decomposition of the lignin and formation of soluble sodium silicate;

(2) Addition of calcium chloride with an appropriate Ca/Si ratio to obtain calcium silicate.

(3) Hydration of calcium silicate and precipitation as calcium silicate hydrate (C–S–H).

Focusing on the inorganic precipitate (P), several studies reported on the use of micro- and nano-sized inorganic particles of C–S–H, distributed as a suspension during the preparation of cement mixtures with water, as promoter of the early stages of setting and hardening of cementitious phases. Cement is a hydraulic binder that develops its mechanical features (shape, toughness and compactness and adhesion) upon complex reactions of dissolution/hydration and precipitation of calcium silicates and aluminate hydrates after mixing the cement powder with water. These steps can be significantly modified by possible heterogeneous precipitation mechanisms driven by the presence of particles acting as seeds in the aqueous fraction. The observed effects involve the rate of hydration (related to the setting and hardening), and the compactness of the material, that shows a higher toughness and a reduced porosity.^19–24^ Further important advantages are the reduced sensitivity to environmental degradation processes, such as freeze–thaw action, salt aggression and carbonation.

Among inorganic industrial products, cementitious materials rank first in terms of product volume. According to the Technology Roadmap for Low-Carbon Transition in Cement, global cement demand is expected to increase to 1600 kg per capita by 2050.^25^ Cement is the second most consumed material worldwide, first being water. Improving the sustainability of cementitious materials involves various strategies, with the use of alternative, waste, and recycled materials throughout the entire production chain being crucial to this effort. In a Portland cement alite (tricalcium silicate – C_3_S‡‡In the cement chemistry, the following abbreviations for oxides are used: C = CaO; S = SiO_2_; A: Al_2_O_3_; F = Fe_2_O_3_; H = H_2_O; C̄ = CO_2_.) is the main hydraulic phase, accounting for more than 60% of the clinker composition, and is responsible for early- and mid-term strength development. Other mineral phases include belite (dicalcium silicate, C_2_S), contributing to late strength improvement, and tricalcium aluminate (C_3_A), playing a crucial role in setting. A well established and effective strategy to improve early strength of cements, acting on silicate phases, is the use of inorganic silicate seedings. As a matter of fact, alite hydration process starts from the outer layer of the grain and propagates toward the centre until complete transformation of C_3_S into C–S–H. Inorganic calcium silicate seeds act as nucleation centres for the growth of silicate hydrates and facilitate the early development of C–S–H needles.

According to Garbev *et al.*,^26^ C–S–H based additives can be activated by a thermal treatment, thanks to the formation of amorphous phases that are particularly reactive in the rehydration process.

This study will evaluate the potential for promoting heterogeneous precipitation of hydrated silicate phases and improving the polymerization degree of silicate hydrates in a cementitious paste. This will be done by using inorganic species produced from the treatment of rice husk. Specifically, the evaluation will involve using a fraction obtained directly through chemical extraction of silicates from rice husk, as well as using this fraction after thermal activation at either 350 °C or 650 °C for 1 hour. The features of the materials obtained by the chemical procedure and the effects of the additives on the cement hydration reactions have been studied using a multi-technique approach based on PXRD, XRF, TGA/DSC, Electron microscopy and NMR.

## Experimental

### Materials

Rice husk is obtained after the drying of the paddy rice and the husking process. Rice husk in this study was provided by a rice production plant in Morano Po (Vercelli, Italy) and was characterized, before its processing by PXRD, TGA, XRF and solid-state NMR to understand its composition.

Cement CEM I 42.5 R, supplied by Buzzi SpA, was used for the nucleation and compressive strength tests. Its phase composition is reported in Table S1.†

All other reactants were purchased from Sigma Aldrich and used without further purification.

### Methods

#### Rice husk treatment

**Fig. 1:**
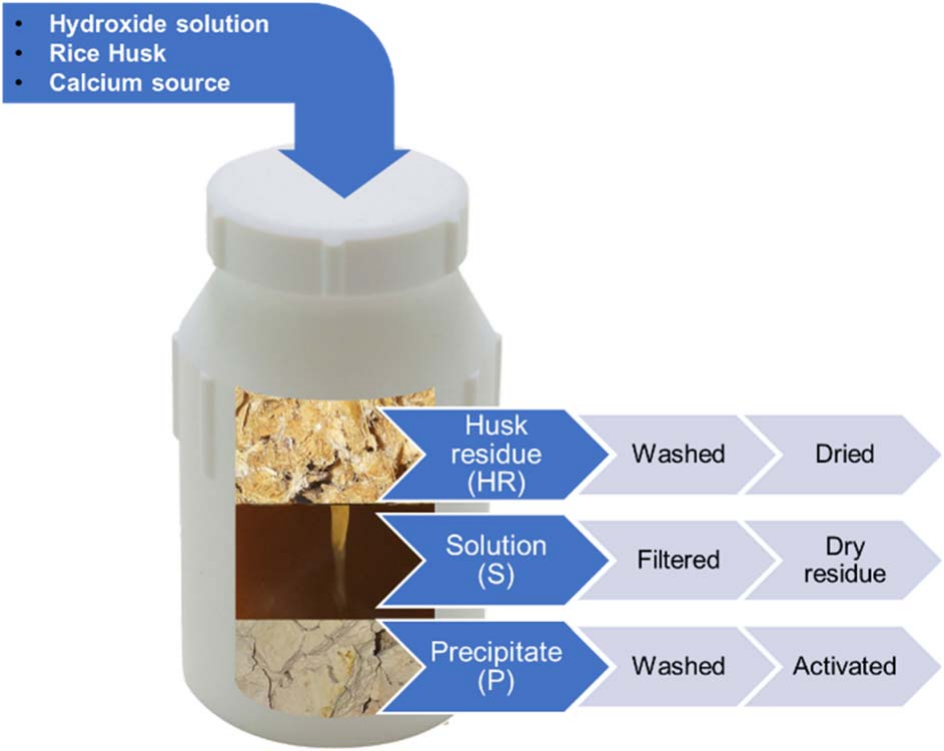
A schematic representation of the chemical process for the valorisation of rice husk based biomass.

A schematic representation of the chemical process is shown in Fig. 1.

As a general procedure, after 30 minutes of stirring, a Ca^2+^ source (CaCl_2_)^27,28^ was added in different proportions, defined with respect to the silica content of rice husk by the Ca/Si ratio. The stirring was then carried out for another hour under N_2_ flow to avoid the precipitation of calcium carbonate. After saturating the solution and headspace with N_2_ the vessel was sealed and treated under hydrothermal conditions at 125 °C for 24 hours.

A heterogeneous mixture of three phases was obtained after the treatment. At the bottom, a beige precipitate was found, along with a brownish fibrous fraction consisting of husk residues, and a brown-ochre solution (see Fig. 1). The three phases were separated, obtaining three samples from each experiment. The husk residue (HR) was removed by sieving the mixture with a coarse mesh sieve and subsequently washed and dried. The precipitate (P) was separated from the liquid by filtering, then washed until neutrality and dried in an oven at 105 °C. A fraction of the solution (S) was dried, and the dry residue collected and weighted to determine the amount of dissolved solids. HR was then washed very abundantly with water to remove precipitate residues and until neutrality.

#### Extraction optimisation

Rice husk, without any prior physical treatment was introduced in a 500 mL Teflon sealed vessel containing 250 mL of sodium hydroxide (NaOH) solution.

The tuning of the chemical procedure has considered the following parameters:

– NaOH concentration

– Amount of biomass

– Ca/Si molar ratio

The amounts and concentrations of reactants used in the experiments are listed in Table 1.

**Table 1:** List of the extraction experiments and conditions

Experiment number	NaOH (mol L^−1^)	Rice husk (g)	Ca/Si ratio	Samples produced
1	2.50	10	2	HR1, P1, S1
2	2.50	13	2	HR2, P2, S2
3	2.50	17	2	HR3, P3, S3
4	2.50	20	2	HR4, P4, S4
5	2.50	10	0	HR5, S5
6	2.50	13	0	HR6, S6
7	2.50	17	0	HR7, S7
8	2.50	20	0	HR8, S8
9	0.50	10	0	HR9, S9
10	2.50	10	1	HR10, P10, S10
11	1.25	10	1	HR11, P11, S11
12	1.25	10	2	HR12, P12, S12

Samples obtained from each experiment were named with the code of the fraction and experiment number (*e.g.* P1, HR1, S1, P2 and so on).

##### Larger scale preparation of P

On the basis of the characterization of the recovered fractions (see Results and discussion section), a larger (40 g) amount of husk was treated according to the conditions of experiment 11, increasing the amounts of water and all other reactants accordingly to produce an adequate amount of precipitate (P11b) to be used in subsequent applicative characterizations and tests.

##### Preparation of cement additives from precipitate samples

Thermal treatments on sample P11b were performed as reported in literature by Garbev *et al.*^26^ heating the sample at 350 and 650 °C for 1 h in order to activate it. The cement additives obtained were named according to the activation treatment: P_ap_ is the P11b sample used as prepared while P_350_ and P_650_ are P11b treated at 350 and 650 °C respectively.

##### Preparation of samples for nucleation/precipitation tests in cements

This procedure was carried out to evaluate the capability of the additive to be dispersed in water and to promote the precipitation of hydration phases in cementitious mixtures. Cement was mixed in a ratio 10 : 1 with the additive P_ap_, P_350_ or P_650_ and water was added in a ratio 100 : 11 to obtain samples WCP_ap_, WCP_350_ and WCP_650_ respectively. Another sample was prepared mixing water and cement only (WC), to be used as a reference. The mixtures were stirred for 2 h, and the powders recovered by filtration and dried in an oven at 40 °C for 20 h.

##### Mortar and paste samples preparation

Mixtures of cement, sand, water (technically named as cement mortars) and additive were prepared according to UNI-EN 196/1 using a Hobart mixer as described in Table 2. P_ap_ is mixed with water and dispersed using the Branson 450 Sonifier. Ultrasonication power was set at 450 W and treatment time was of 20 minutes. P_350_ and P_650_ were instead mixed with the dry powders directly, then hydration water was added.

**Table 2:** List and description of the mixtures prepared for the mechano-physical tests

Mixture	Amount of cement (g)	Sand (g)	Additive	Amount of additive (g)	Amount of water (g)
CEM	450	1350	—	—	225
CEM-P_ap_	450	1350	P_ap_	4.5	225
CEM-P_350_	450	1350	P_350_	4.5	225
CEM-P_650_	450	1350	P_650_	4.5	225

The limited amount of precipitate at our disposal allowed to prepare only 9 cubes sized 40 × 40 × 40 mm (instead of prisms sized 40 × 40 × 160 mm as prescribed by UNI EN 196-1). Compressive strength measurements were performed after 16 h, 1 d, 2 d, 3 d and 7 d of aging using a mechanical press. Each measurement was performed twice except the last one (after 7 d) which could be measured only once.

Samples for the hydration kinetic study were prepared using the same method and composition reported in Table 2 but without adding the sand, obtaining therefore cement pastes instead of mortars. These samples were named CEP, CEP-P_ap_, CEP-P_350_ and CEP-P_650_ according to the type of additive used.

#### XRF

Prior to the XRF analysis, the rice husk was calcined to eliminate the organic fraction, and fused beads were prepared mixing calcined samples with Li-tetraborate 1 : 10 using a standard fluxing procedure for ceramic materials. X-ray fluorescence analysis was performed using a Panalytical Axios spectrometer. IQ+ semi-quantitative software was used for the data treatment allowing the determination of the element content (expressed as a percentage by weight of the corresponding metal oxide).

#### PXRD

X-ray powder diffraction patterns were obtained on a ThermoARL XTRA48 diffractometer using Cu Kα radiation (*λ* = 1.54062 Å). All powder diffraction spectra were measured in continuous mode using the following conditions: 2*θ* angular range 2–70° for standard measurements; tube power 45 kV and 40 mA, step size 0.02° 2*θ*, scan rate 0.5° min^−1^.

#### TGA/DSC

Thermogravimetric analysis (TGA) was performed on a Mettler Toledo TGA/DSC 1 instrument under air or inert gas (N_2_) flow, with a gas flow of 20 mL min^−1^. The samples were heated in the variable range from 35 to 950 °C with a rate of 20 °C min^−1^. Thermograms were corrected by subtraction of the background curve, obtained without sample in the same experimental conditions.

#### SEM/EDX

SEM images at different magnification were recorded on a Quanta 200 FEI Scanning Electron Microscope equipped with EDAX EDS attachment, using a tungsten filament as electron source at 25 keV. For the deposition, sample solutions in ethanol were prepared and for 20 minutes, then deposed on the hot Al stub with an airbrush. The so-prepared samples were coated with 45 nm of gold.

#### ssNMR

Solid-state NMR spectra were acquired on a Bruker Avance III 500 spectrometer equipped with a wide bore 11.75 Tesla magnet with operational frequencies for ^1^H, ^13^C and ^29^Si of 500.13, 125.77 and 99.35 MHz, respectively. A 4 mm triple resonance probe with MAS was employed for all experiments. Samples were packed on a Zirconia rotor and spun at a MAS rate of 10–15 kHz. The magnitude of the radio frequency fields was 100 and 42 kHz for ^1^H and ^29^Si NMR experiments, respectively. All ^29^Si MAS NMR spectra were recorded under high-power proton decoupling conditions while the ^13^C NMR spectra were recorded using CPMAS experiments. The relaxation delays, d1, between accumulations were 60, 5 and 5 s for ^29^Si, ^13^C and ^1^H NMR, respectively and chemical shifts are reported using *δ* scale and are externally referenced to TMS at 0 ppm.

^29^Si MAS NMR offers quantitative information on the fractions of silicon found in various tetrahedral environments in a cement paste, labelled as *Q*^*n*^(*m*Al), with (0 ≤ *n* ≤ 4) and (0 ≤ *m* ≤ *n*). Here, ‘*Q*’ denotes a silicate tetrahedron linked *via* oxygen bridges to both aluminium and a combination of ‘*n* − *m*’ silicon tetrahedra. We computed the degree of hydration (DOH), the mean chain length (MCL) and the ratio of aluminium to silicon (Al_IV_/Si) in C–S–H/C–A–S–H on the basis of the deconvolution of the signals from the ^29^Si NMR spectra using the equations reported in the literature.^29,30^

Degree of hydration (DOH), obtained with the formula:DOH = *Q*^1^ + *Q*^2^ + *Q*^2^(1Al) + *Q*^3^

Mean Chain Length (MCL) calculated with the following formula:
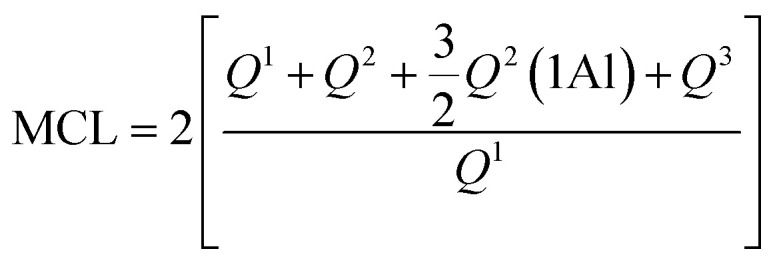


Al^IV^/Si ratio calculated using the following equation.
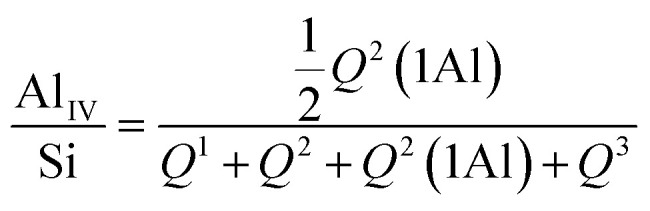


#### FT-IR

ATR-IR spectra were collected on a Fourier transform infrared (FTIR) Nicolet 5700 spectrometer (Thermo Optics) at a resolution of 4 cm^−1^ in the spectral range from 4000 to 400 cm^−1^ and 128 scans using the Attenuated Transmission Reflectance (ATR) module and microscope bench.

#### Dynamic light scattering (DLS) analysis

Particle size distribution was measured in a water dispersion of P11b by Dynamic Light Scattering (DLS), matching as closely as possible the conditions of cement hydration. For this scope, dispersions of 0.5 g L^−1^ and 1 g L^−1^ of P11b in water were prepared using a Branson Sonifier 450, used at full power (450 W) for 20 minutes. The suspensions were submitted to DLS measurements right after the sonication and after 24 hours using a DLS Zetasizer NanoSeries (Malvern Panalytical).

## Results and discussion

As a preliminary step, rice husk was characterized to assess its composition and especially the silica content. The powder X-ray diffraction pattern of raw rice husk is shown in Fig. S1 top,† and exhibits several broad peaks at around 16°, 23° and 35° 2theta values and are associated to crystalline cellulose structure.^31^ Furthermore, the broad feature in the 10–50° 2theta values are related to the presence of amorphous components such as hemicellulose and lignin^32,33^

Elemental composition expressed as oxides and in relative wt percentage after calcination and fusion in borate glass beads (see “Materials and methods” for details) are reported in Table S2.† The composition is typical of a natural-based matrix, as indicated by the presence of the soil elements. The powder X-ray diffraction (PXRD) analysis of the calcined rice husk (Fig. S2†) highlights the presence of cristobalite phase (a polymorph of quartz) resulting from the densification of the silica fraction during its thermal treatment. The formation of dense silica phases during the thermal treatment of rice husk is often cited as a significant drawback. This issue arises both from burning husks in open fields and in industrial plants. The presence of fine, dense silica particles in the fumes can pose health risks if inhaled, potentially leading to lung diseases.^34^

**Fig. 2:**
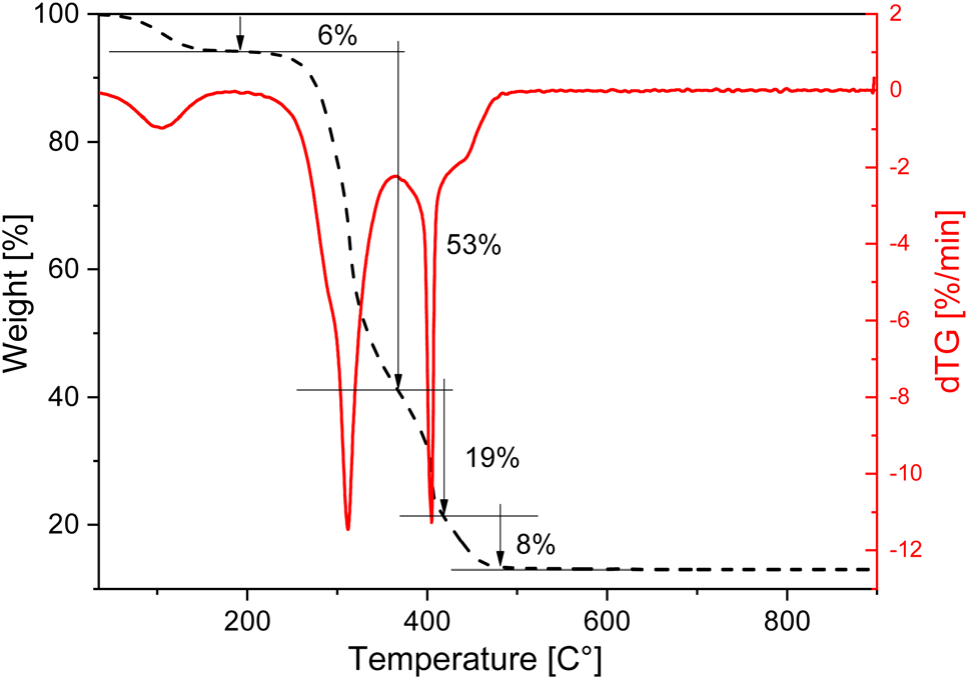
TGA and dTG curves of raw rice husk recorded under air flow.

The thermogravimetric profile of the rice husk was measured under air flow from room temperature (RT) to 900 °C to evaluate its thermal stability and decomposition profile and is shown in Fig. 2. Four main weight loss steps can be seen in the curve. The first loss, at low temperature, is due to the removal of physisorbed water and is followed by two weight losses of 53% and 19%, occurring between 280 and 450 °C, related to the decomposition of the organic components, such as cellulose, hemicellulose and lignin. The dTG curve highlights that the larger weight loss, centred at 311 °C, shows a low combustion rate compared to the one at 400 °C, indicating that the sample under exam does not contain a large amount of volatile compounds. The high combustion rate of the third loss is instead typical of rice husk with a low lignin content.^15,16^ By combining the XRF results to the TGA analysis the SiO_2_ content in this rice husk can be estimated around 13%. This is a bit lower than values reported in the literature, as it is often reported that the content can vary according to genotypes of rice, which ranges between 22 and 15%.^5,8,35^

**Fig. 3:**
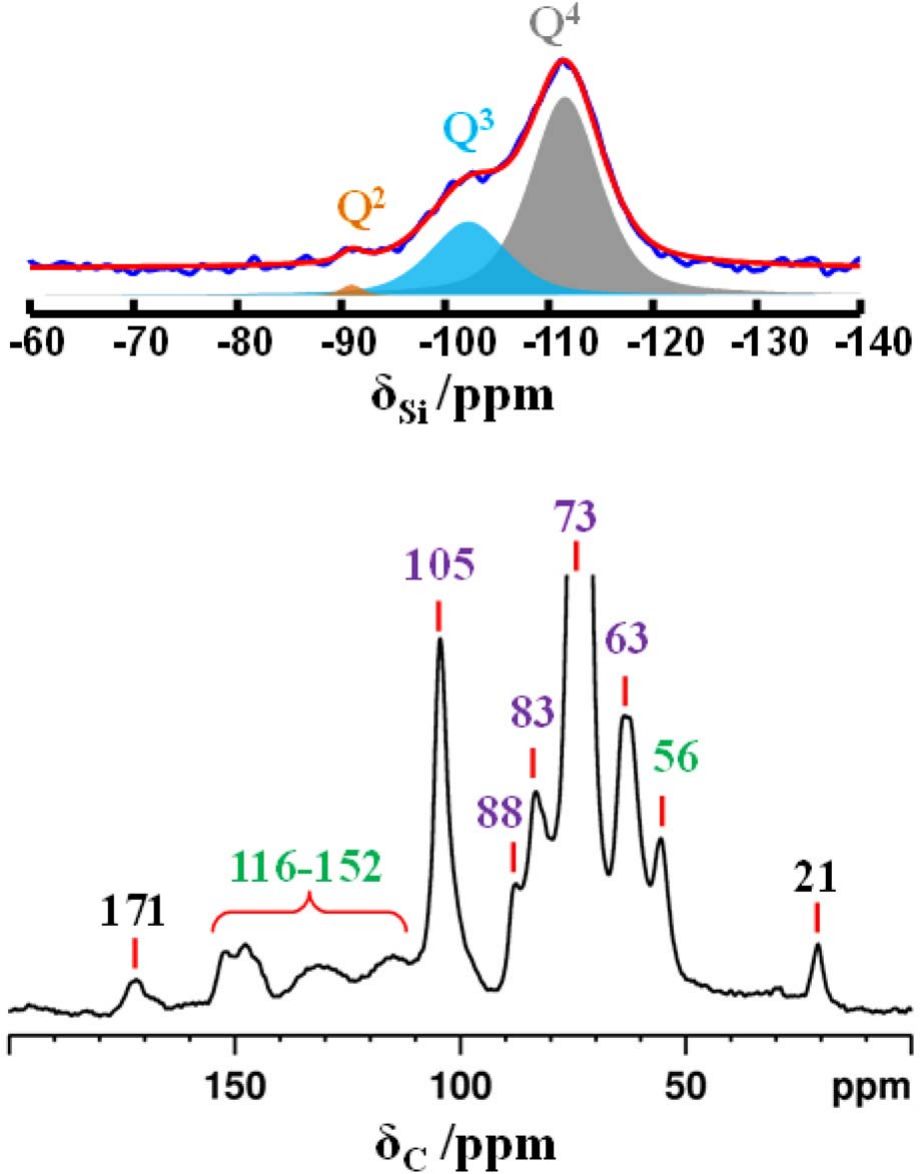
^29^Si MAS (top) and ^13^C CPMAS (bottom) NMR spectra of raw rice husk. In violet, the signals of cellulose, in black those of hemicellulose, and in green those of lignin.

Solid-state NMR spectroscopy was exploited to study simultaneously the amorphous and crystalline components in solid samples.^36^ It provides different spectral insights through the observation of multiple nuclei in the same solid sample that can help to collect structural, chemical and dynamics information. Based on the high silica content in raw rice husk, the ^29^Si MAS NMR spectral data was collected to define the silicon coordination environments (Fig. 3, top). There are three Si sites that were detected as very broad peaks in the raw rice husk sample, namely, *Q*^2^, *Q*^3^ and *Q*^4^ sites. The very broad nature of the resonance peaks is a clear indication of the amorphous nature of the silica present in rice husk.^37^ Consequently, the highly polymerized state of silica is established by the higher amounts of *Q*^4^(Si(OSi)_4_) silicon site at around −110 ppm and *Q*^3^(Si(OSi)_3_OH) site at around −100 ppm. The organic components of rice husk were analysed using ^13^C CPMAS NMR spectroscopy, and the resulting spectrum is presented in Fig. 3, bottom. ^13^C resonance peaks corresponding to cellulose, the primary organic constituents of rice husk, were observed at the following chemical shifts; 63, 73, 83, 88 and 105 ppm. The signals at 21 and 171 ppm are associated with the acetate groups of hemicellulose (with some overlap with cellulose carbons). Finally, the signals at 56 ppm as well as those in the range between 116 and 152 ppm (aromatic carbons) are attributed to lignin.^37^ The high intensity of the ^13^C peaks in the NMR spectrum, attributed to cellulose and hemicellulose, is indicative of their abundance in the rice husk matrix.

### Extraction procedure and experimental parameters optimisation

In this section, details on the results of the experiments performed according to Table 1 to optimise the chemical process will be given, mainly based on the evidence from different characterisation techniques applied on the recovered fractions.

As a general overview, in the first experiments (1–4) the amount of rice husk is increased and the mass of CaCl_2_ used is changed in order to keep the Ca/Si ratio constant. The experiments 5–9 were instead performed without adding the Ca source and changing the amount of husk or NaOH to study the effect of the alkaline treatment on the degradation of the organic part. The last experiments were performed reducing the Ca/Si ratio according to what emerged from the analysis of samples from previous experiments. For each experiment, heterogeneous mixtures are obtained consisting of the fibrous solid residue from the rice husk (HR), a brownish solution (S) which contains both organic components of the husk and possibly reactant residues and other minerals extracted from the rice husk. When CaCl_2_ is added to the reactants, a beige precipitate (P) is found on the bottom of the vessel.

Each experiment, according to Table 1, produces two (when the calcium source is not added) or three samples, marked in the text with the fraction code and experiment number (*e.g.* HR1, P2, …).

The efficiency of the different extraction conditions has been evaluated considering (i) the efficacy in the degradation of the organic fraction, (ii) the silica extraction yield, (iii) the precipitate quantity, and (iv) the purity of C–S–H *vs.* other unwanted phases (evaluated from the PXRD measures).

**Fig. 4:**
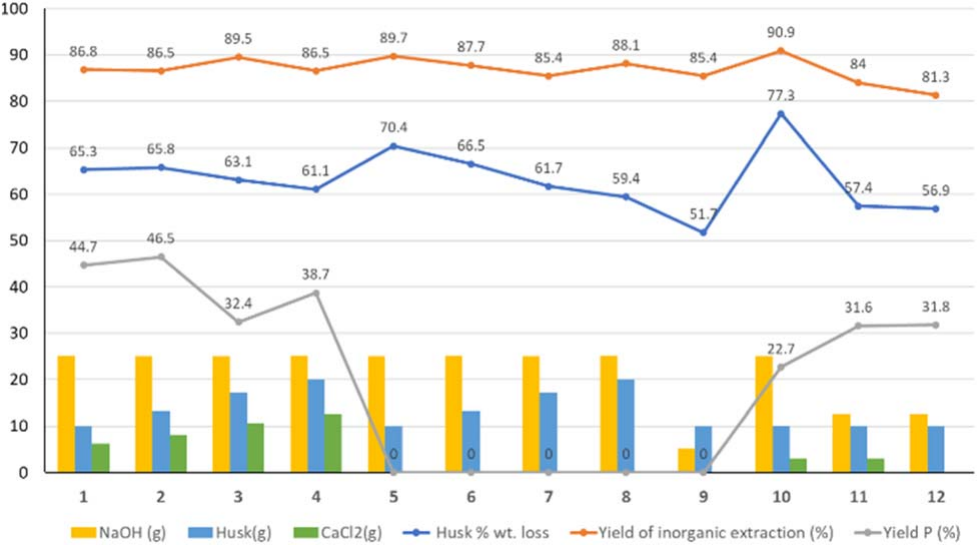
Yields of the experiments. Experiment number on the *x* axis. The bars represent the amount of reactants, the lines represent the yields of the reaction.

The weights of the recovered fractions (after washing and drying in oven at 65 °C) and of the HR fractions after calcination were measured, and indices for the first three benchmark key aspects were calculated (Fig. 4).

The three benchmark parameters are husk % wt loss (calculated on the amount of organic fraction obtained after the treatment with respect of the starting rice husk quantity), the yield of inorganic extraction (obtained as the weight of HR after calcination *vs.* the weight of the starting husk after calcination), and the yield of precipitate (P) % (calculated using the weight amount of precipitate over the rice husk quantity).

Keeping the ratio between Ca and Si constant and increasing the amount of husk (experiments 1–4), the husk % wt loss due to the extraction procedure, used to evaluate the overall inorganic extraction and organic degradation, shows values around 65% and it decreases when the amount of husk increases (in experiments 1 through 4 and 5 through 8 without CaCl_2_), probably due to a less efficient mixing during the procedure. The calcination of HR leads to a residue (mainly composed of silica) that allows calculating the residual % of inorganics after the extraction procedure and, by comparing it to the initial inorganic amount in the husk (13,8%), the yield of inorganic extraction. These yields are generally over 86% and appear to be linked to the amount of NaOH. The yields of P were then calculated with respect to the initial mass of husk, and they vary between 23 and 47%. In experiments 3 and 4 the large amount of P produced prevented a complete recovery through filtration, leading to an apparently smaller yield. Since the precipitate is likely not of pure C–S–H, it is not possible, from these data alone, to calculate a yield of conversion of the silica content into C–S–H.

Experiments 5–8 were performed without adding CaCl_2_ and varying the amount of husk to study the effect of the extraction procedure on the organic part by comparing them pairwise with experiments from 1 to 4. The amount of HR increases linearly with the increase of the husk and is very similar to that of experiments from 1 to 4. The extraction yields are all similar, indicating that the amount of residual inorganic in the fibrous fraction is not affected by the presence of CaCl_2_ in solution. Based on the setup, the amount of 10 g of husk was chosen as optimal and used for subsequent experiments.

Experiments 9–12 were performed by fixing the amount of husk at 10 g during the synthesis and varying the amounts of NaOH and CaCl_2_. Comparing the results of experiment 9 and experiment 5 reveals that decreasing the molarity of the NaOH solution reduces the efficiency of the extraction procedure. The presence of increasing amounts of CaCl_2_ in the solution seems to accentuate this effect since the decrease of NaOH and increase of CaCl_2_ in experiments 11 and 12 lead to higher HR amounts and lower extraction yields.

**Fig. 5:**
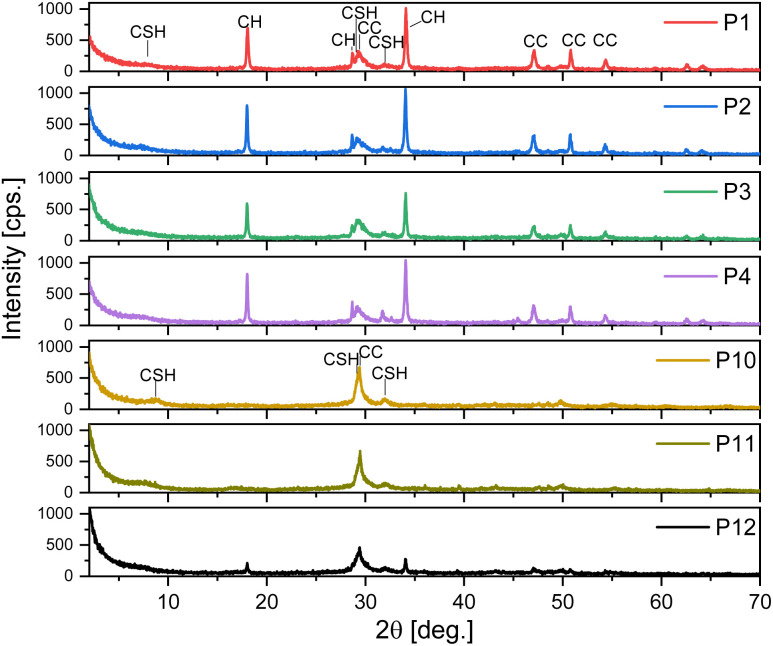
PXRD patterns of the precipitates (P1–P4, P10–P12).

A large amount of precipitate does not necessarily imply the success of the reaction, since other inorganic phases, which are not of interest, can precipitate together with C–S–H. Therefore, all P samples, were analysed by PXRD (Fig. 5) and phase identification was performed. Despite a low crystallinity, C–S–H is identified by two broad peaks: the basal peak at 8–9° (*d*-spacing 11.32–10.14 Å) and the one at 32° (2.78 Å) and marked as CSH in Fig. 5. The position of the basal peak of C–S–H is influenced both by the presence of water and the Ca/Si ratio, as larger *d*-spacing are due to a lower Ca/Si ratio and lower hydration.^38^ C–S–H phases are also characterized by an intense peak at 29.3° (3.04 Å) that is superimposed to a calcite peak. These peaks are clearly visible in the precipitates from experiments 10 and 11 (marked P10 and P11) and less intense in the other samples. Notably, the obtained C–S–H shows some degree of crystallinity while the C–S–H phases, most commonly found during the hydration of Portland-type cementitious materials, are mostly amorphous. In samples P1, P2, P3, P4 and P12 other peaks are present, indicating that the extraction procedure leads to the formation of other phases, all compatible with the cement chemistry. Peaks marked as CH are due to Ca(OH)_2_ (portlandite structure). Calcite (CaCO_3_), originated by carbonatation of CH due to CO_2_ in the atmosphere, is also present, and its peaks are marked as CC. The formation of these phases could compete with C–S–H since they limit the availability of calcium ions. The CH and CC phases are particularly abundant in the precipitates from the first four experiments (from P1 to P4) and are almost absent in P10 and P11, that contain only some calcite, as indicated by the presence of the peak at 29.36° but no portlandite.

These results indicate that lowering the amount of CaCl_2_ (*i.e.* Ca/Si ratio) leads to almost the same amount of precipitate with low calcite content and more crystalline C–S–H, avoiding the precipitation of excess calcium in the form of portlandite.

**Fig. 6:**
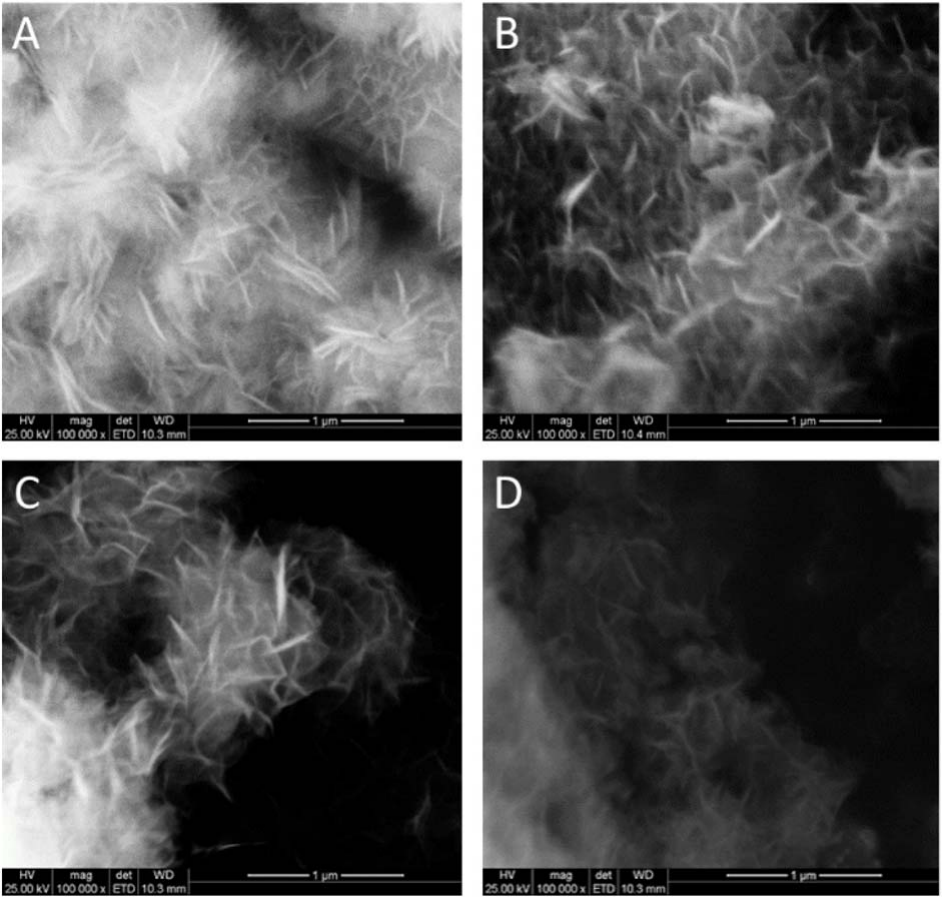
SEM images of the precipitates: (A) P1, (B) P10, (C) P11, (D) P12.

SEM images of the precipitates (Fig. 6) show the typical morphology of C–S–H, formed by delaminated layered aggregates composed of sheets about 250–260 nm long. The morphology of the precipitate and dimension of the sheets is very similar in all samples; therefore, it does not seem affected by the amount of NaOH or CaCl_2_ used during the extraction process. The micrometric dimension of the aggregates and the porosity are promising features for the application of the precipitate as additive in cementitious materials since a high surface area favours the reactivity.

#### FT-IR

FT-IR spectra collected on the precipitates P1, P2, P3 and P4 (Fig. S3†) reveal the presence of C–S–H by the very intense band at 960 cm^−1^ attributed to the asymmetric stretching of Si–O of *Q*^2^ silica groups and by the intense band around 820 cm^−1^ attributed to Si–O stretching of *Q*^1^ silica groups.^39^

Moreover, the broad band in the 3600–3050 cm^−1^ region is attributed to loosely hydrogen-bonded interlayer water. The sharp peak at 1645 cm^−1^ is attributed to the presence of portlandite Ca(OH)_2_. The numerous other bands are instead due to the presence of organic phases.

With respect to samples P1, P2, P3 and P4 (Fig. S3†) precipitates P10, P11 and P12 (Fig. S4†) show similar C–S–H signals but an increasing reduction of the peaks related to the organic fraction (upon decreasing the NaOH concentration). This confirms that the reduction of the amount of NaOH limits the amount of organic components that contaminates the precipitate. The intensity of the band at 960 cm^−1^ is similar in P10, P11 and P12 while in the last two samples the band at 820 cm^−1^ is less intense than in P10, indicating a lower amount of *Q*^1^ silica. The band attributed to the presence of hydrogen bonded interlayer water (in the 3600–3050 cm^−1^ region) is less intense in P11 and P12 indicating a lower water content in these precipitates. This could indicate a lower Ca/Si ratio in these samples.^39^ The band at 1645 cm^−1^ is absent in P10 and barely visible in P11 and P12 confirming that the amount of Ca(OH)_2_ is lower than in P1, P2, P3 and P4.

FT-IR spectra measured on the HR samples are reported in Fig. S5–S7.† In these spectra we see a series of signals that can be globally attributed to cellulosic components, as reported in several literature references. The OH stretching region can be influenced by inter- and intra-molecular hydrogen bond vibrations in cellulose^40^ and can suggest a denser or looser organic structure upon the treatment. Also the absorption bands at 1428, 1367, 1334, 1027 cm^−1^ and 896 cm^−1^ belong to stretching and bending vibrations of –CH_2_ and –CH, –OH and C–O bonds in cellulose.^41,42^ On the basis of the complexity of the spectra in the 700–1000 cm^−1^ region, a straightforward confirmation of the presence of the silicate fractions (by stretching vibrations around 1030 cm^−1^ and 770 cm^−1^) cannot be obtained. Notably, the organic moieties signals are lower in the HR10 than in the HR11 and HR12 spectrum. Summarising all the evidence, experimental conditions 10, 11 and 12 seem to produce the purest samples of precipitate (see P samples infrared spectra in S4 and HR spectra in S7). They therefore represent a good compromise between the extraction of silica and the purity of the precipitate and suggest that the best extraction conditions are the ones used in experiment 11 that, moreover, use a lower amount of NaOH.

### Analysis of samples from the optimized synthesis conditions

A larger amount of sample for subsequent tests the extraction procedure was obtained by repeating the procedure, with the conditions of experiment 11, on 40 g of husk. Recovered products, namely-precipitate (P11b), husk residues (HR11b) and solution (S11b) were fully characterized.

### Analysis of the precipitate (P11b)

The PXRD pattern of P11b reported in Fig. S7† is very similar to that of P11 indicating that the scale-up did not affect the extraction results from the viewpoint of the obtained crystalline phases.

**Fig. 7:**
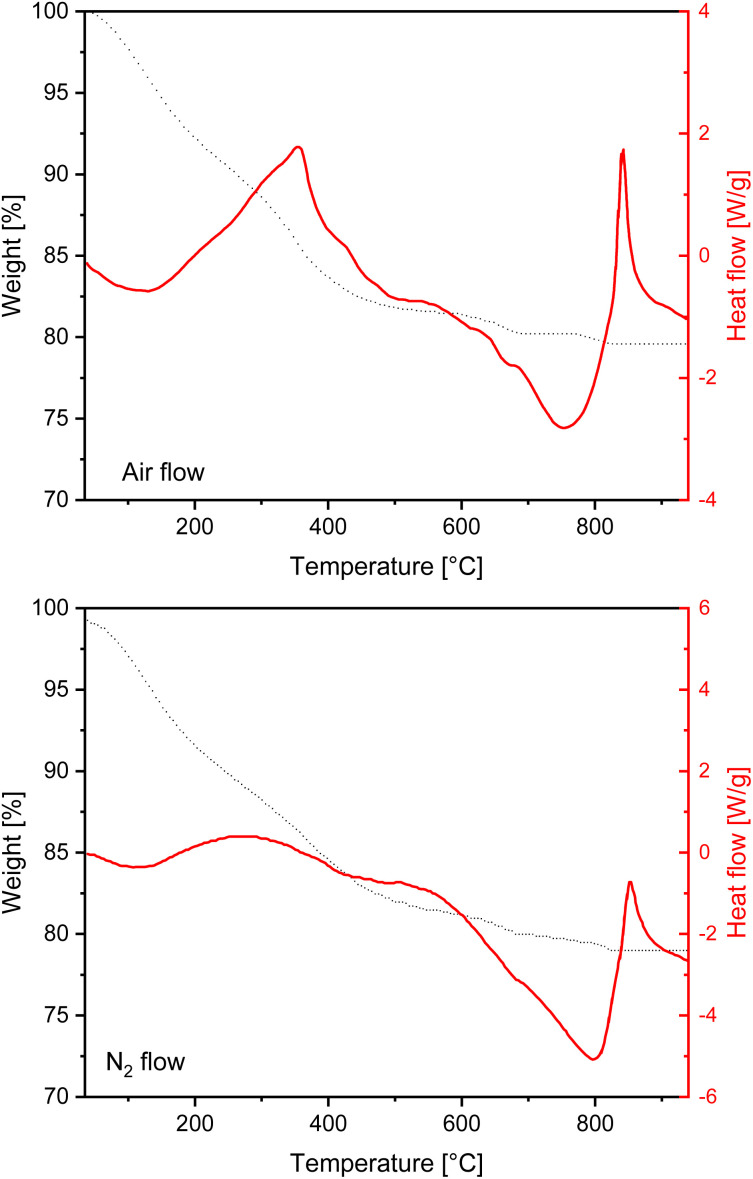
TGA (black dotted line) and DSC (red continuous line) curves of P11b measured under air flow (top) and under nitrogen flow (bottom).

The TGA-DSC profile of P11b recorded both under air and inert flow shows a 7.8% wt loss between 150 and 330 °C r (Fig. 7 top), with an exothermic peak in air (see DSC curve) due to the combustion of organic residues in the precipitate. Repeated measurements on different extraction experiments showed some differences in the organic content calculated by combustion, with a data spanning around 5–8% wt. The weight loss of the 0.6%, occurring above 700 °C and in correspondence with an endothermal peak in the DSC curve, is due to the decomposition of CaCO_3_ to CaO. The elemental analysis of the sample was performed by SEM/EDX using a mapping procedure over portions of about 15 μm^2^ on different points (on different particles) of the sample. The average % composition is reported in Table S3.† By observing the data we can see that all Ca^2+^ provided in the reaction mixture is retrieved in the precipitate (as also verified by the complexometric titration of Ca^2+^ in sample S11b, that did not contain any Ca^2+^ in appreciable amounts). The EDX results on sample P11b highlight the absence of other elements from the starting material and a very low content of alkali metals K and Na, of about 2.7 and 0.3% respectively, and about 3% overall.

### Analysis of the husk residue (HR)

**Fig. 8:**
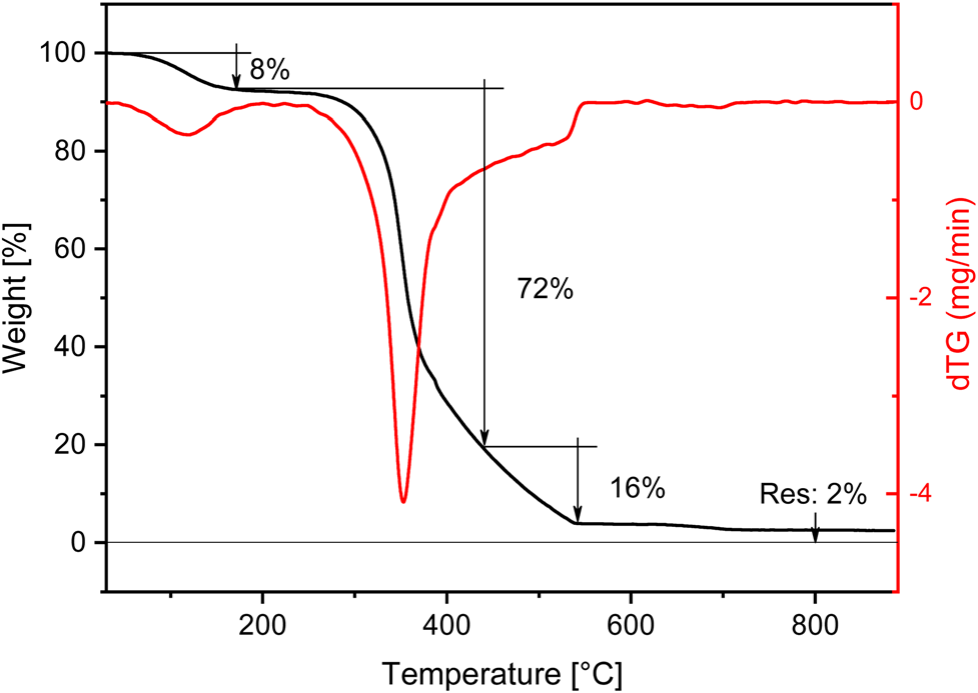
TGA of sample HR11b (after washing) measured under air flow.

The TGA of HR11b (Fig. 8) measured after very abundant washing with deionised water, under air flow, shows an almost complete removal of the silica fraction originally present in rice husk. The first weight loss of the 8% wt can be ascribed to physisorbed water. Then, a large weight loss, spanning over 300 to 550 °C, with a cumulative weight loss of about 88% wt, can be observed, due to the combustion of the organic part. At 650 °C a 2% weight loss due to the decarbonation of residual CaCO_3_ brings the residue, attributed to residual silica in the treated husk, to less than 2% wt.

**Fig. 9:**
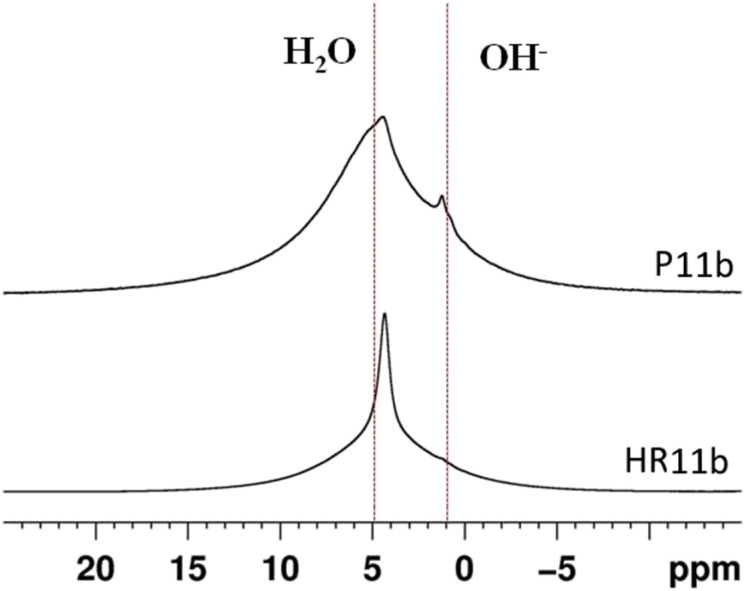
^1^H MAS NMR spectra of P11b and HR11b.

^1^H MAS NMR spectra (Fig. 9) of HR11b shows a sharp peak around 6 ppm, while in P11b the same signal is broadened. This signal is attributed to water, which is present as free water in HR11b and as bonded structural water in P11b. Weak bands at around 1 ppm, due to the hydroxyl groups of calcium hydroxide and silanols, are also present in P11b.

**Fig. 10:**
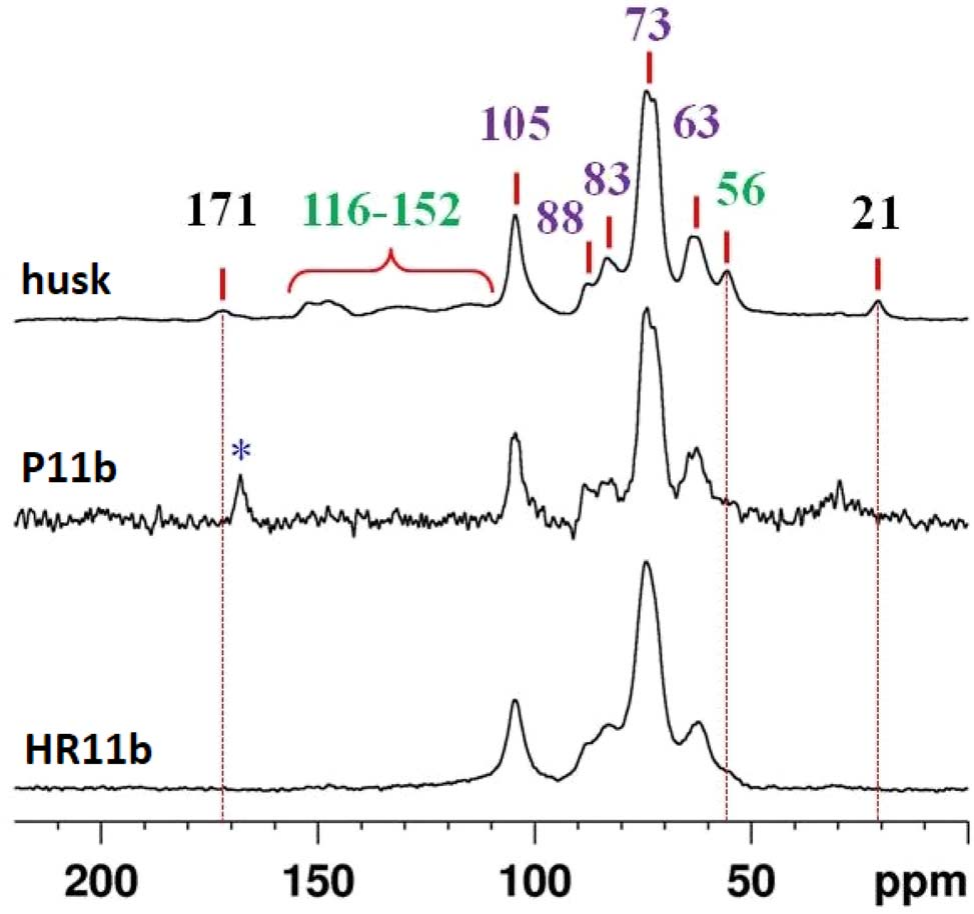
^13^C CPMAS NMR spectra of rice husk, P11b and HR11b.

The ^13^C CPMAS NMR spectra of P11b and HR11b are reported in Fig. 10 in comparison with that of the untreated rice husk. Signals attributed to lignin and hemicellulose in the husk (labelled in green and black respectively), are not present anymore in HR11b and in P11b, suggesting that they have been successfully hydrolysed by the alkaline treatment. The cellulose signals (labelled in violet) are still visible in HR. Accordingly, this fraction, with a very low amount of silica content will be addressed in forthcoming studies, through hydrolysis by enzymatic digestion, to sugars extraction and to the production of bio-ethanol *via* alcoholic fermentation. Another application under study is to add it directly to the polymer melt to produce polymer based composite materials. A fraction of cellulose is also retrieved in P11b, although much less than in HR11b. A signal at 168 ppm (highlighted by *) is consistent with a fraction of calcium carbonate in the form of calcite.

### Characterization of the precipitate for the application as nucleation additive in concrete

To characterize the suitability of P11b as nucleating agent for cements, its hydrodynamic particle size and capability to operate as nucleating agent have been evaluated.

Dynamic Light Scattering (DLS) measures on the dispersion in water of P11b show that, when the concentration of P11b is 1 g L^−1^ (Fig. S9†), there are three components in the dispersion right after sonication (red curve), the first one with an average radius of 100 nm, another around 600 nm and the third one at 3000 nm which is cut by the upper instrumental limit. After 24 h (green curve) the coarser particulate has deposited, and the suspension mainly contains particles with a radius around 300–400 nm. The same test was repeated with a concentration of 0.5 g L^−1^ (Fig. S10†). This time, right after the sonication, the size distribution of the particles in suspension has a single peak around 350 nm, indicating a more homogeneous size distribution and a smaller average size than in the 1 g L^−1^ suspension, and after 24 h (green curve) the intensity of the peak has diminished but the maximum has not shifted. The increase of the width at half maximum of the peak indicates that spontaneous aggregation of the precipitate may have occurred.

### Characterization of P after thermal treatment

A thermal treatment of P11b was performed at 350 and 650 °C for 1 h in order to activate the precipitate, as reported in literature by Garbev *et al.*,^26^ and the resulting materials were studied by means of PXRD and ^29^Si MAS NMR.

**Fig. 11:**
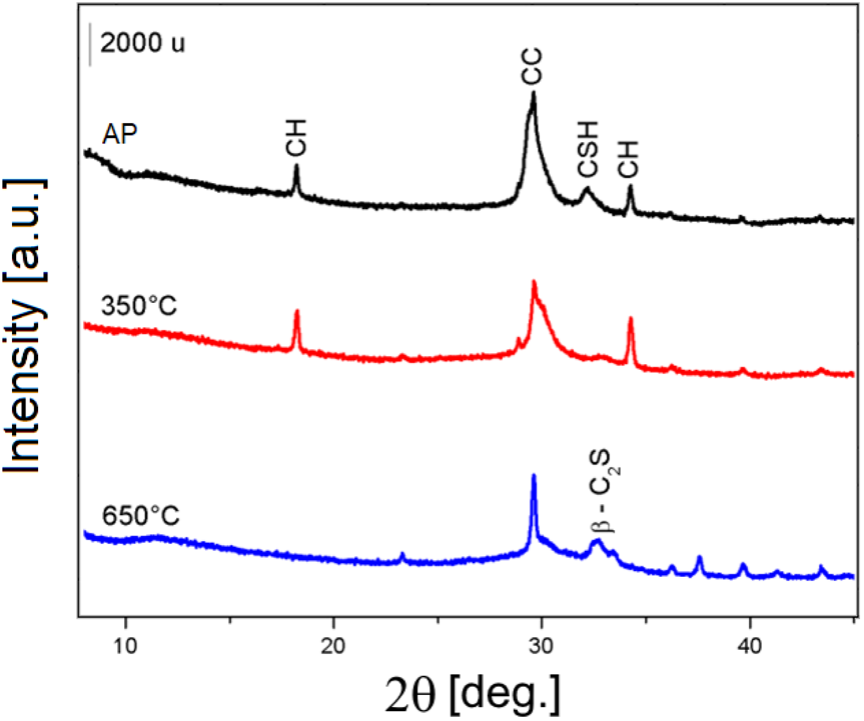
PXRD pattern of P11b as prepared (P_ap_ – black), after thermal treatment at 350 °C (P_350_ – red), and after thermal treatment at 650 °C (P_650_ – blue). CH = Ca(OH)_2_ portlandite CC̄ = CaCO_3_ calcite; CSH = calcium silicate hydrate; β-C_2_S = anhydrous β-dicalcium silicate.

From the PXRD patterns reported in Fig. 11, it is evident that, with respect of the untreated sample P_ap_, there is a significant decrease in the intensity of the calcite peak at about 30° and that of the C–S–H peak at 32° 2*θ* upon thermal treatment at 350 °C (sample P_350_). Upon thermal treatment at 650 °C (sample P_650_) the portlandite peaks disappear, indicating its decomposition, while anhydrous β-dicalcium silicate is formed, as indicated by the peak marked β-C_2_S.

**Fig. 12:**
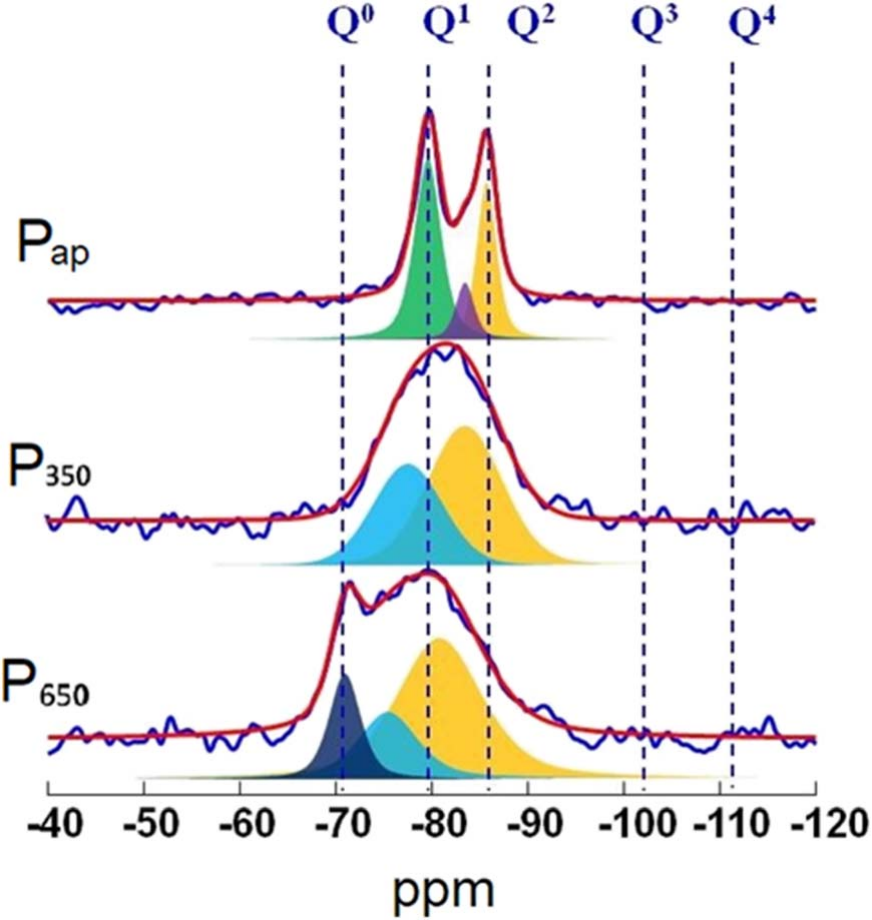
^29^Si MAS NMR spectra of P11b as prepared (P_ap_), after thermal treatment at 350 °C (P_350_), and after thermal treatment at 650 °C (P_650_).

^29^Si MAS NMR spectrum (Fig. 12, top) of P_ap_ shows three relatively sharp peaks at −79, −82.5 and 85 ppm, respectively, due to *Q*^1^, *Q*_b_^2^ and *Q*_p_^2^ (b and p refer to bridging and pair *Q*^2^ silicate units) of a crystalline C–S–H phase. More importantly, no silica peaks (*Q*^3^ and *Q*^4^) are detected in the spectrum confirming its complete conversion to C–S–H phase. Upon thermal treatment at 350 °C, those sharp peaks disappear and very broad peaks, down-field shifted, appear. From these results it can be suggested that the crystalline structure of C–S–H is destroyed upon thermal treatment due to the extraction of structural water resulting in a disordered calcium silicate phase. The ^29^Si NMR spectrum of sample treated at 650 °C showed the emergence of a new, sharp peak at about −71 ppm and is attributed to the formation of β-C_2_S.

Moreover, the detection of broad peaks indicate that the thermal treatment duration has been insufficient for the complete conversion of disordered calcium silicate phase into crystalline β-dicalcium silicate.

### Analysis of the mixtures prepared for the nucleation test

The ability of the precipitate to promote nucleation in cement was assessed by preparing mixtures of water, cement CEM I 42.5 R and additives, as described in the Materials and methods section. These conditions, characterized by an excess of water, were designed to favour crystal growth. Scope of these experiments was to evaluate the effects of the thermal activation of the P11b fraction on its ability to promote the precipitation of hydrated phases in a cementitious mixture upon hydration. The region of interest of the PXRD patterns measured on samples WC, WCP_ap_, WCP_350_ and WCP_650_ after hydration are reported in Fig. S11.† The PXRD patterns of all samples containing the additive show a reduction of the intensity of the C_3_S (alite) peaks, particularly evident in WCP_350_ and WCP_650_, and an increase of the bands of C–S–H (tobermorite) and of CH (portlandite) with respect to the reference.

### Physico-mechanical tests on cement mortars

**Fig. 13:**
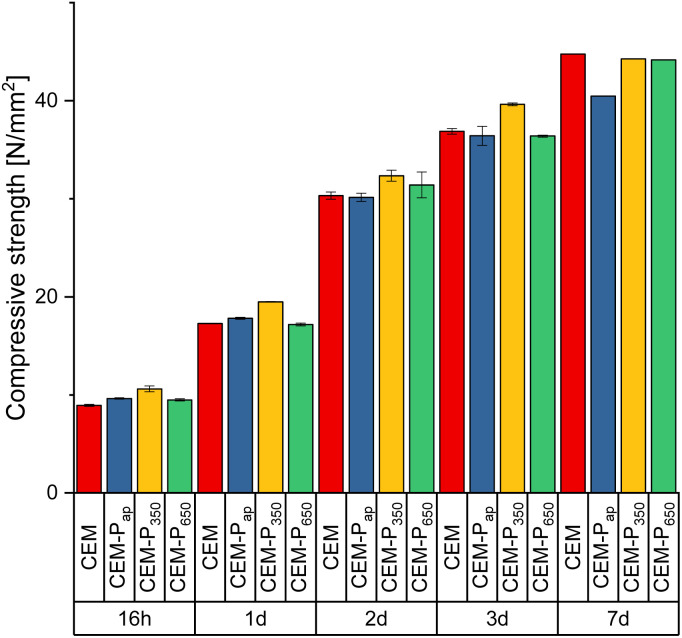
Evolution of the compressive strength of mortar samples with ageing.

The macroscopic effects of the nucleating agents were tested using the precipitate, with or without thermal activation (P_350_, P_650_ and P_ap_) as additive in cement mortars as reported in the Materials and methods section. The results of the compressive strength tests at different aging times are reported in Fig. 13 and the data in Table S4 in the ESI.† Sample CEM-P_350_ shows an increased compressive strength with respect to the reference sample (CEM) at short aging times confirming early strength gains due to the presence of additive. After 7 d of ageing the effect of the additive becomes insignificant. CEM-P_ap_ is similar to CEM at short ageing times and seem to have performances lower than the reference sample at long ageing times. CEM-P_650_ instead does not differ significantly from the reference at any ageing times. This trend can indicate that the additive acts primarily on C_3_S phases, which are solubilized rapidly, but not on C_2_S phases which requires longer times to react.

### Hydration kinetic study of the pastes by PXRD and ^29^Si SS-NMR

**Fig. 14:**
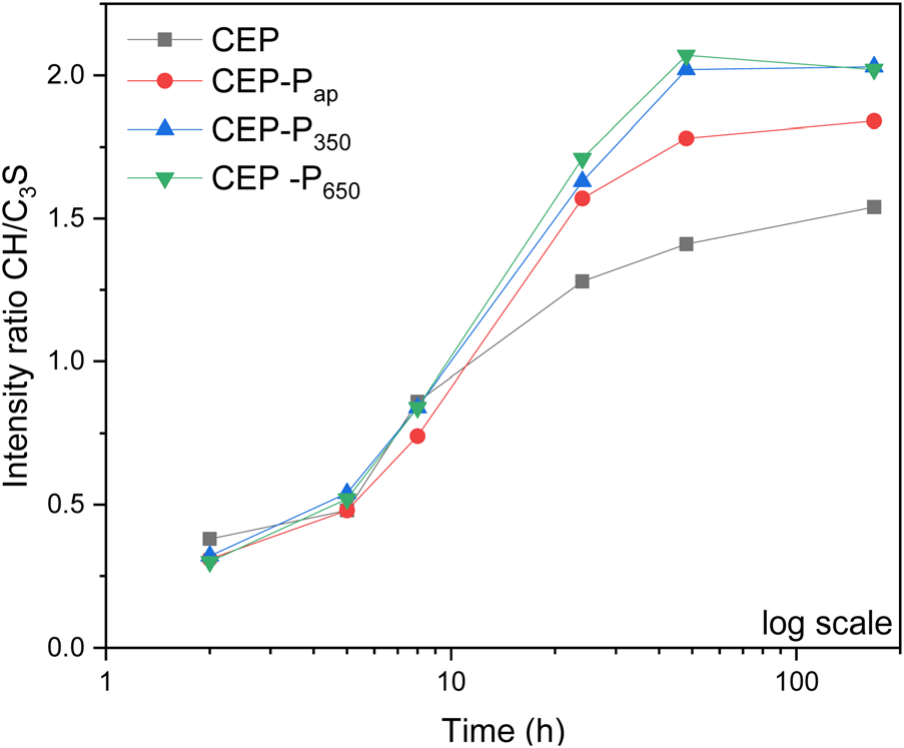
Intensity ratio of the PXRD peaks of CH (34.4°) and C_3_S (34.6°) plotted *versus* time (in log scale).

To shed light on the mechanisms of action of the additive, PXRD analysis was performed *in situ* on cement pastes (CEP, CEP-P_ap_, CEP-P_350_ and CEP-P_650_) prepared according to Table 2. In all samples, the C_3_S peak decreases (see the PXRD patterns reported in Fig. S12†) in favour of the CH peak upon hydration, but in samples with the additive the CH peak is visible after 5 h already, while in the reference sample it starts to be visible after 8 h. The different kinetics induced by the additive is clearly visible in Fig. 14 where the intensity ratio between the CH and C_3_S PXRD peaks *vs.* time is reported. In samples with the additive, the ratio between CH and C_3_S peaks starts to increase earlier and is always higher than in the sample without additive (CEP).

**Fig. 15:**
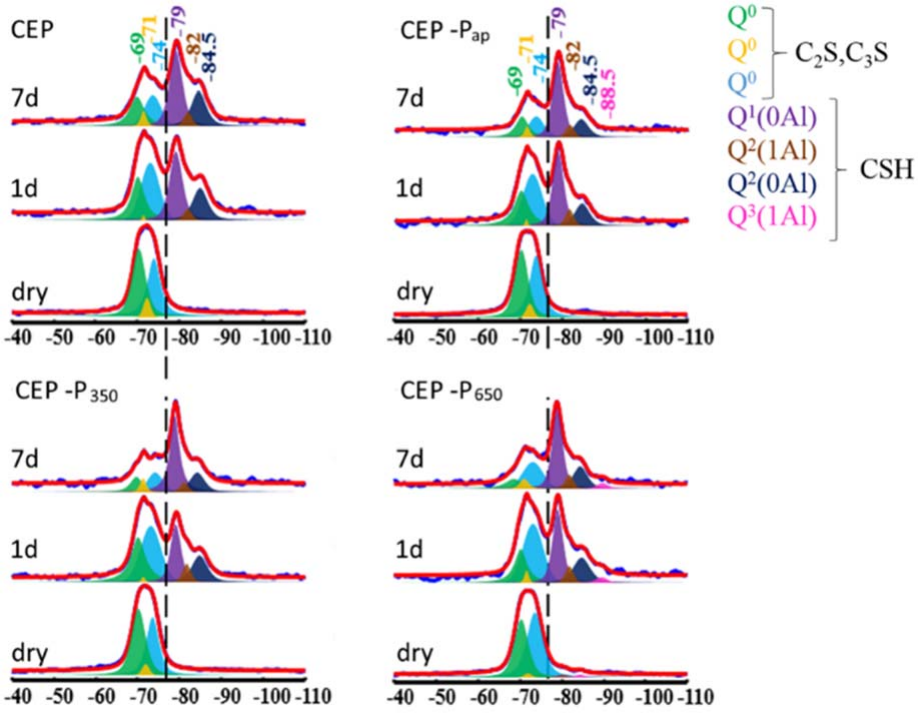
^29^Si SS-NMR spectra measured on the paste samples before hydration, after 1 day and after 7 days.

The hydration of silicate phases was then studied in detail by ^29^Si SS-NMR. ^29^Si SS-NMR spectra (Fig. 15) measured on powders before hydration, after 1 day and after 7 days, show the formation of the same species in all samples, except in sample CEP-P_650_ where the signal *Q*^3^(1Al) = −88.5 ppm (C–S–H) is visible (in pink in Fig. 15). After 1 day the hydrated phases (signals on the right of the dashed line) have similar relative intensity to those of the anhydrous phases (C_2_S and C_3_S) in all samples except CEP-P_350_, where anhydrous phases prevail. After 7 days the amount of hydrated phases is higher in the samples with the C–S–H additive with respect to the CEP sample.

**Fig. 16:**
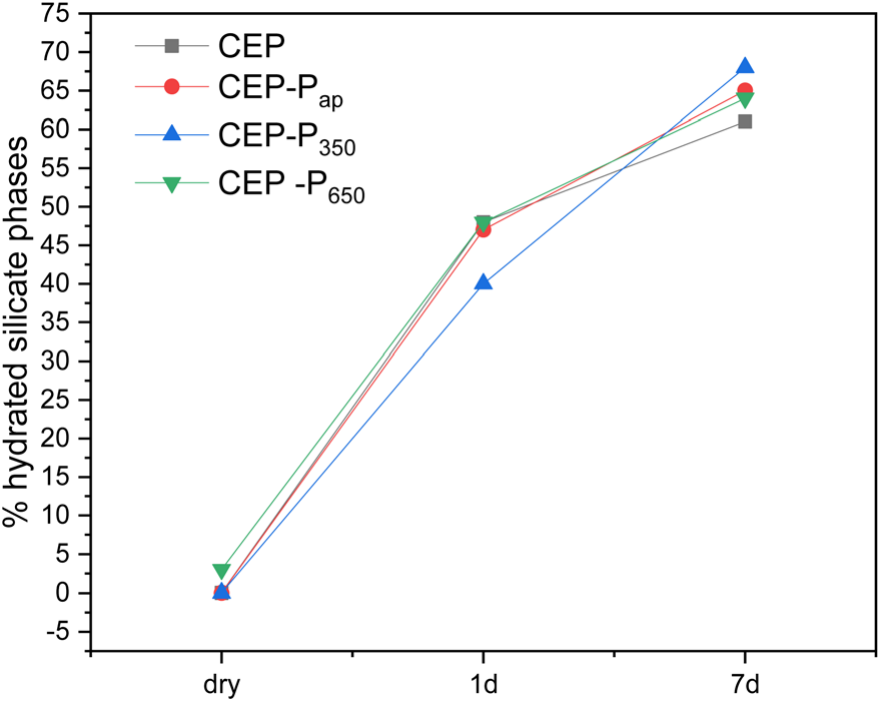
Plot of the % of silicate hydrated phases (C–S–H) on the total silicate phases *vs.* hydration time, from ^29^Si SS-NMR data.

This difference can be better appreciated by looking at the semi-quantitative analysis of the NMR data reported in Fig. 16, where the % of silicate hydrated phases (C–S–H) on the total silicate phases is plotted. Samples CEP-P_ap_ and CEP-P_650_ have a behaviour similar to CEP after one day from the preparation of the paste. The additive P_350_ significantly affects the hydration processes, initially slowing them down. However, after 7 days, the sample CEP-P_350_ exhibits the highest percentage of hydrated phases, exceeding 10%. In contrast, samples CEP-P_ap_ and CEP-P_650_ show a slightly higher percentage of hydrated phases (about 5% more) compared to CEP, but still less than that observed in CEP-P_350_.

More in detail, by the calculation of the parameters describing the conversion of the cementitious matrix upon hydration (see Experimental section for details) reported in Table 3, the following consideration can be outlined.

**Table 3:** Parameters derived from the deconvolution of ^29^Si NMR measurements on cement pastes during hydration (estimated error ± 5%)

Sample	DOH	MCL	Al^IV^/Si
CEP-HY1d	48	3.48	0.031
CEP-HY7d	61	3.40	0.033
CEP-P-HY1d	47	3.30	0.053
CEP-P-HY7d	65	3.09	0.046
CEP-P_350_-HY1d	40	4.05	0.062
CEP-P_350_-HY7d	68	3.0	0.037
CEP-P_650_-HY1d	48	3.85	0.042
CEP-P_650_-HY7d	64	3.43	0.047

According to these results, it clearly appears that the presence of the additive affect significantly the Al^IV^/Si ratio in C–A–S–H, increasing it in general with respect of the cement alone.

This evidence is consistent with a role of the P additive on the phases most quickly reactive in the cement (*i.e.* with the first 24 hours) essentially related to aluminate phases (tricalcium aluminate, C_3_A and tetracalcium ferroaluminate C_4_AF). The role of the additive seems to be different on the basis of the temperature of treatment. P_350_ is the most effective in including Al in the hydrated calcium silicate gel (as shown by the highest Al^IV^/Si ratio), promoting a higher mean chain length even limiting the hydration of silicate phases present in the material, as suggested by the lowest DOH. The presence of a highly polymerized C–A–S–H can be consistent with a tougher network structure consistent with an improved mechanical performance (*vide infra*).

Aside, between 1 and 7 days the rate of hydration is very high, as the sample reaches a value of 68 compared to reference sample CEP-HY7d, producing a further diffuse formation of C–A–S–H with a low chain length, resulting in a mean value of 0.037.

With respect of P350, the additive treated at 650 °C (P650) seems to be less effective in modifying these parameters, such as DOH and MCL with respect to reference material. In the latter material, evidences from PXRD and SS-NMR show the formation of a silicate phase, dicalcium silicate C_2_S, typically present in an ordinary Portland cement, that is known to have a slow hydration rate in cementitious pastes.

By comparing the results of PXRD and SS-NMR on the paste samples to the results of the compressive strength tests it can be deduced that the compressive strength developed after 16 and 24 hours can be partially related to the amount of hydrated phases, but most relevantly to an extended and well-formed C–A–S–H gel structure due to the inclusion of Al in the hydrated silicate gel. This resulting network structure is commonly addressed in the literature as toughening the material, and positively affecting the porosity of the matrix.^20^ In the 1–7 days hydration period, the samples with P additives show a relevantly higher extent of hydration with respect to the reference, and this can be consistent with the increase of mechanical resistance. So far, these findings would require a dedicated and systematic study on the effect of the obtained additives since the preliminary results are promising.

## Conclusions

A single step eco-efficient mild chemical process for the conversion of rice husk based biomass is reported here and is optimized by tuning the pH and Ca/Si ratio in favour of the precipitation of silicate hydrate phases (C–S–H), which can be used as a nucleation additive in cements. The process maximizes the extraction of silica from the husk, whose presence makes the use or even the disposal a challenge. The described chemical process can be extended to any straw and husk based cereal crops (wheat or barley) after optimization of the Ca/Si ratio. The precipitate, rich in C–S–H, was characterized by PXRD, SS-NMR, TGA, SEM and FT-IR spectroscopy and determined its composition and morphology. It was tested as a possible nucleation additive in cements and obtained promising results. In fact, it appears to contribute positively to the early nucleation of hydrated cement phases, improving its mechanical properties. Moreover, the treatment proved to be effective in promoting the delignification of the husk and the complete removal of the silica, as observed by the PXRD and SS-NMR analysis of the husk residues (HR). The results from PXRD, ^29^Si NMR and mechanical test suggest that the most effective additive is obtained after a thermal treatment of the precipitate fraction at 350 °C.

A future work will explore several promising applications of the remaining two fractions studied. The fibrous organic phase will be tested as a filler in polymers to assess its potential benefits. The liquid fraction, which is rich in polysaccharides and mineral salts,^43,44^ could potentially be used as a plant supplement following neutralization of its high pH and substitution of NaOH with KOH (a manuscript on this topic is currently under preparation). Additionally, we will investigate the feasibility of reusing this liquid to repeat the extraction process, aiming to enhance the sustainability and efficiency of the method.

## Data availability

The data supporting this article have been included as part of the ESI.†

## Author contributions

Conceptualization EB, DG, MM; data curation EC, VG; formal analysis: EC, VG; funding acquisition EB, EC; investigation VT, GP, MM; methodology EB, DG; project administration EB, VG; resources EB, EC, DG; supervision EB, VG, DG; validation VT, VG, GP, MM; visualization EC, GP, VT; writing – original draft VT, EB, GP; writing – review & editing EC, VG.

## Conflicts of interest

There are no conflicts to declare.

## Supplementary Material

RA-014-D4RA05263C-s001
