# Supplementary material for: Single step eco-efficient mild chemical process for the total valorisation of rice husk: a focus on the inorganics as a cement additive
Source: RSC Adv. 2024 Nov 13;14(49):36314–26. doi: 10.1039/d4ra05263c (PMC11558517; doi:10.1039/d4ra05263c)
Supplement: RA-014-D4RA05263C-s001 [file RA-014-D4RA05263C-s001.pdf]

Supplementary Information (SI) for RSC Advances.
This journal is © The Royal Society of Chemistry 2024

Electronic Supplementary Information

Single step eco-efficient mild chemical process for the total valorisation of rice husk: a focus on inorganics as cement additives

Eleonora Conterosito, Geo Paul, Valentina Toson, Valentina Gianotti, Marco Milanesio, Daniela Gastaldi, Enrico Boccaleri

Phases	CEM I 42.5 R
C_3S	66.9
C_2S	7.2
C_3A	13.7
C_4AF	3.6
Gypsum	3.6
Other	5

Table S1. Phase composition of CEM I 42.5 R

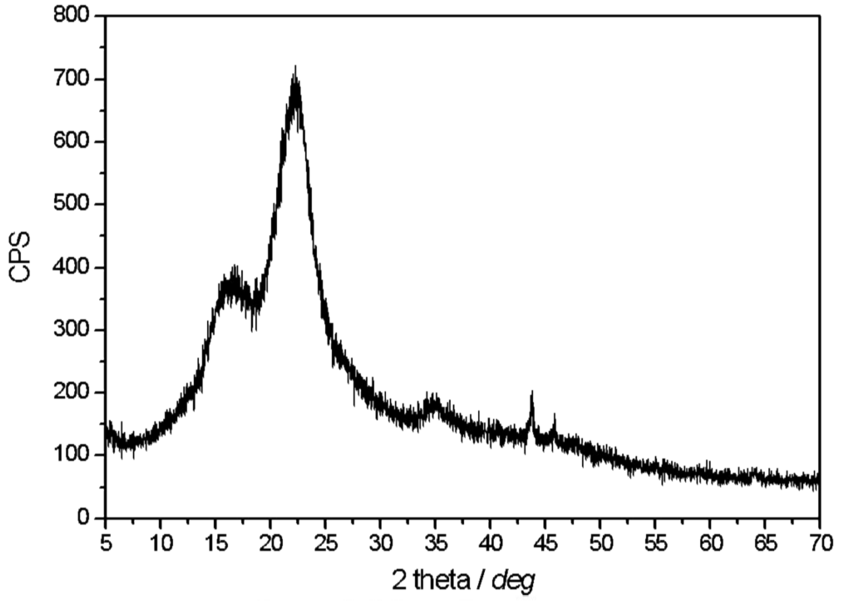

Figure S1. Powder X-ray diffraction of raw rice husk.

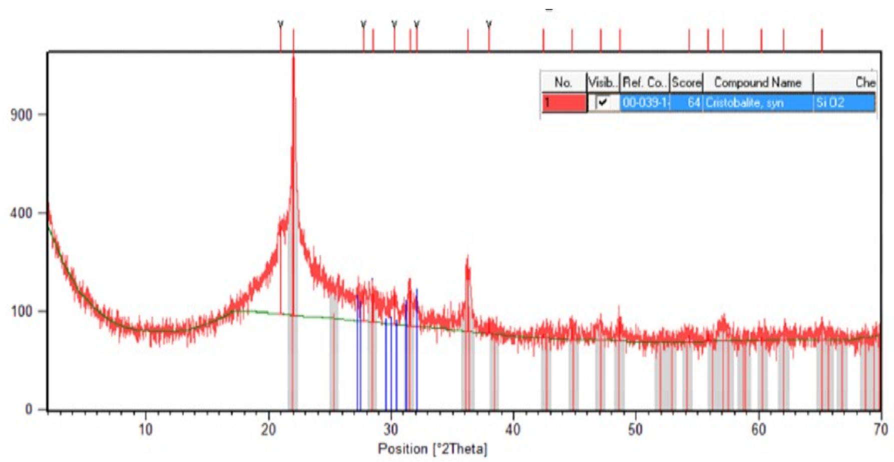

Figure S2. Powder X-ray diffraction of calcined rice husk and phase matching with cristobalite.

Oxide	Wt%
SiO_2	85,64
K_2O	4,14
CaO	4,05
SO_3	1,40
P_2O_5	1,02
Fe_2O_3	0,96
Cl	0,84
Al_2O_3	0,74
MnO	0,59
MgO	0,37
Na_2O	0,08
TiO_2	0,06
TOTAL ($SiO_2 + Al_2O_3 + Fe_2O_3$)	90.65

Table S2. Chemical composition of rice husk as determined by X-ray fluorescence analysis after calcination and expressed as % in weight (average error estimated ± 2%).

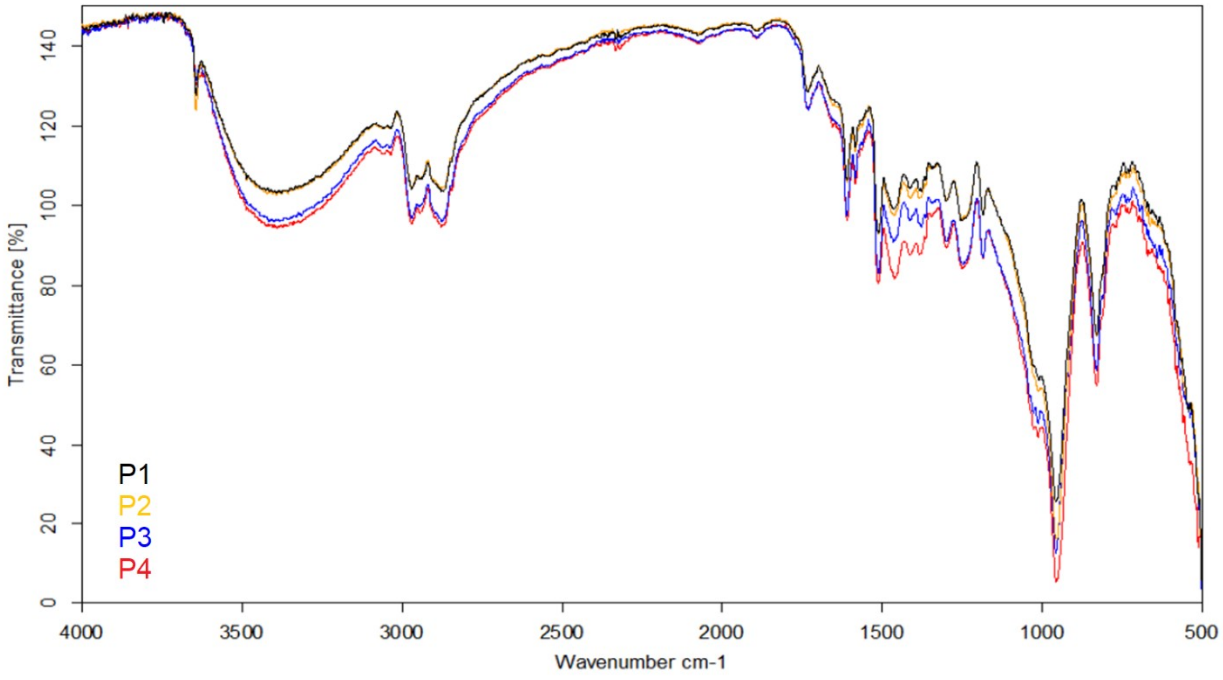

Figure S3. FT-IR ATR spectra of the precipitates: P1 (black), P2 (yellow), P3 (blue), P4(red).

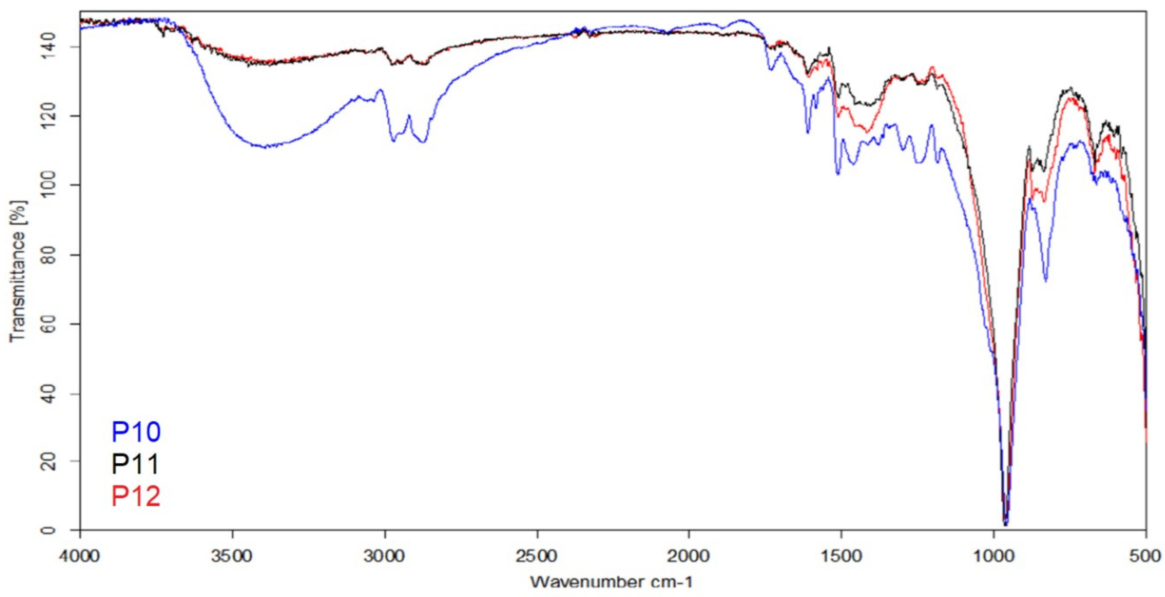

Figure S4. FT-IR ATR spectra of the precipitates: P10 (blue), P11(black) P12 (red).

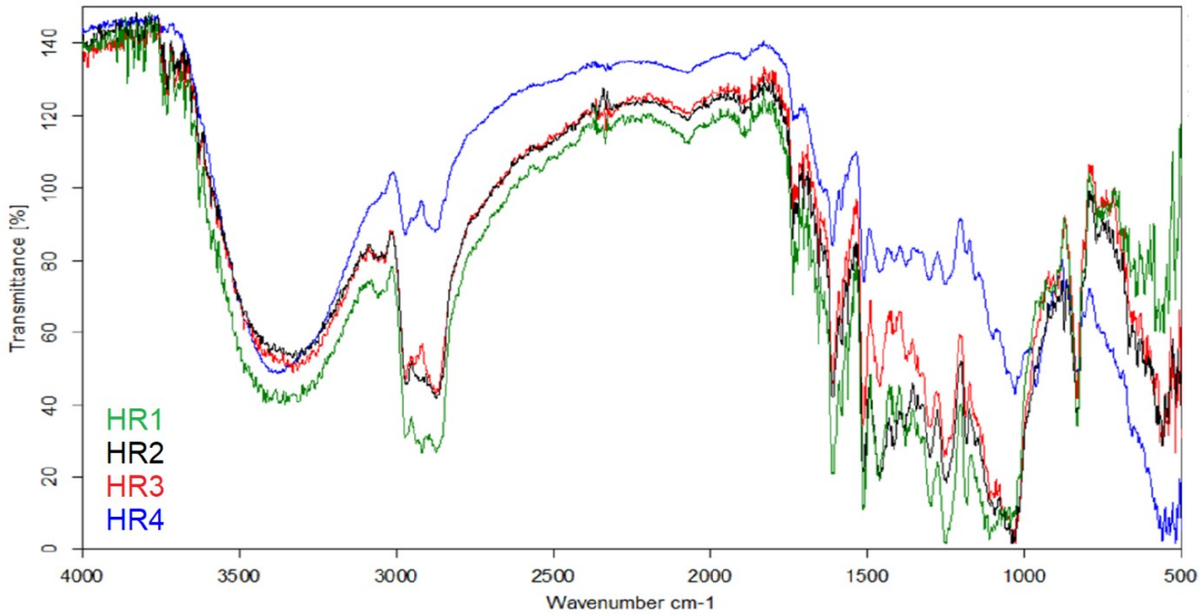

Figure S5. FT-IR ATR spectra of the husk residues: HR1(green), HR2 (black), HR3 (red), HR4 (blue).

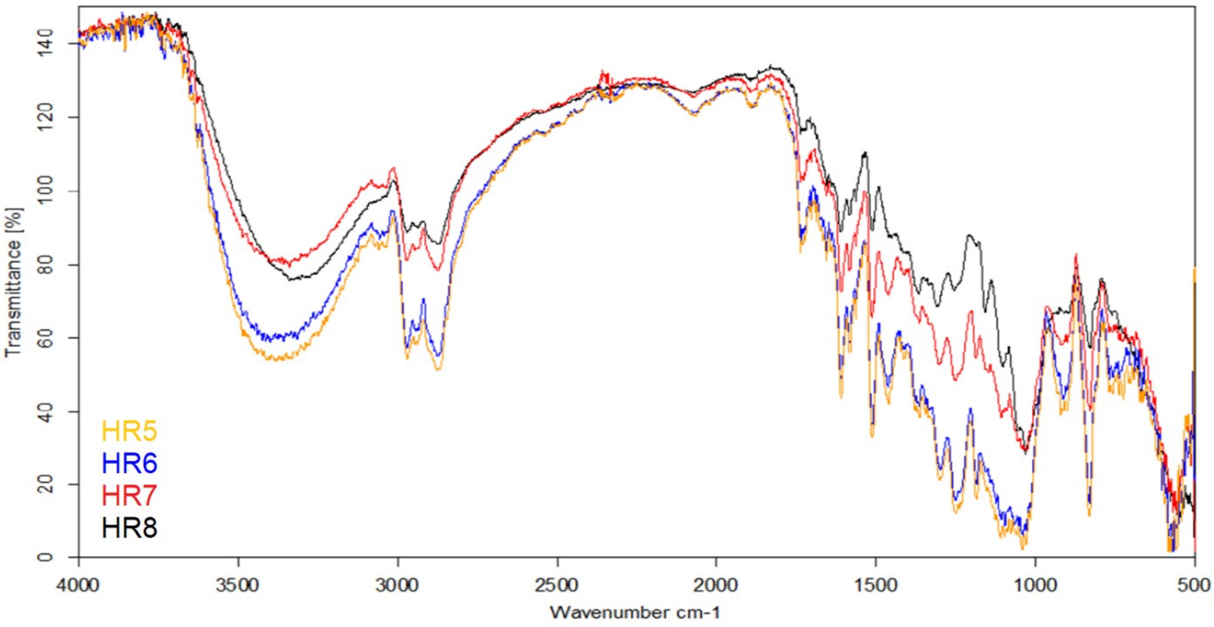

Figure S6. FT-IR ATR spectra of the husk residues: HR5(yellow), HR6 (blue), HR7 (red), HR8 (black).

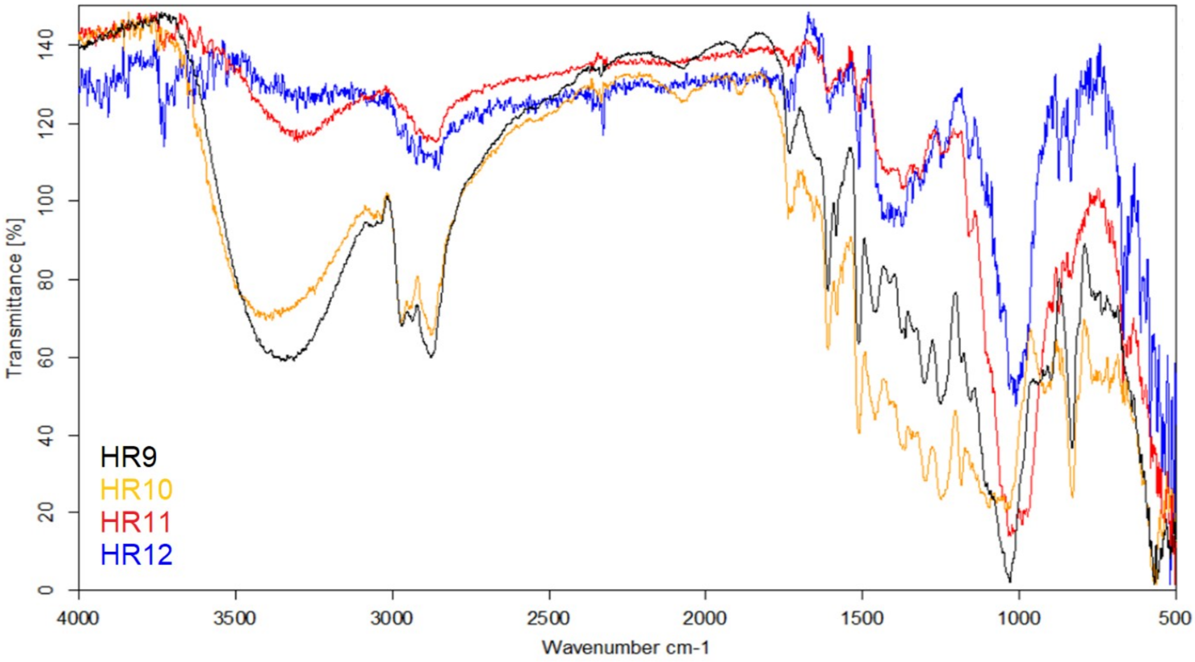

Figure S7. FT-IR ATR spectra of the husk residues: HR9(black), HR10 (yellow), HR11 (red), HR12 (blue).

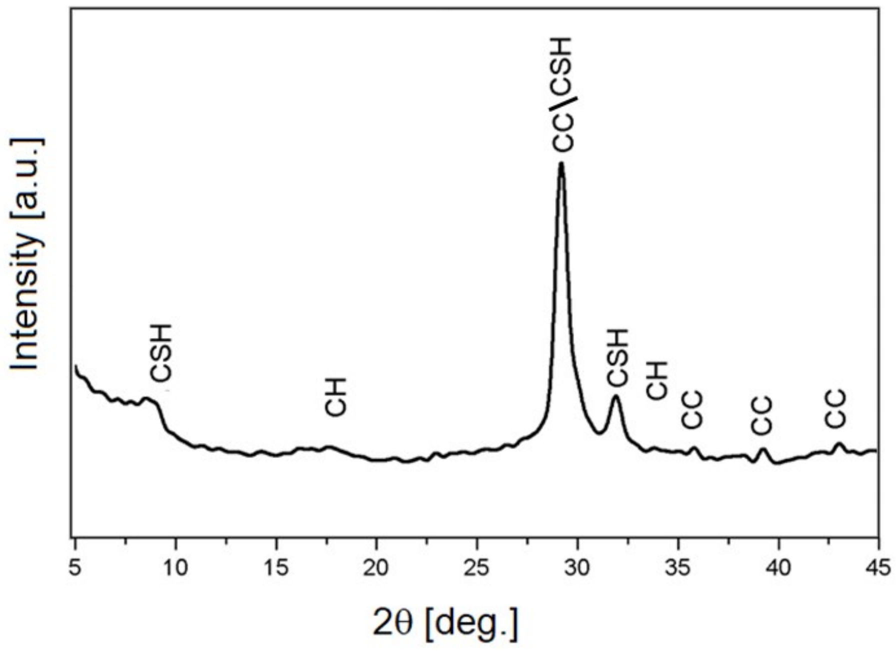

Figure S8. XRPD pattern of **P11b.**

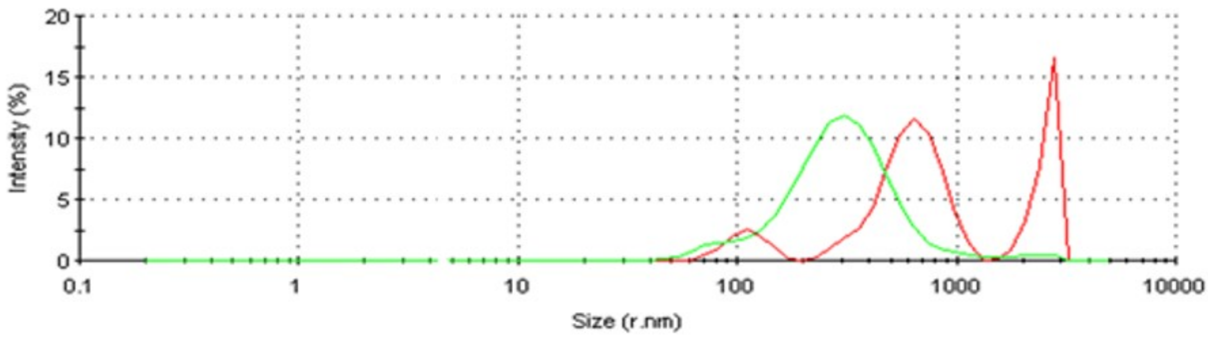

Figure S9. Size distribution by intensity from DLS analysis on the water dispersion prepared using a concentration of precipitate of 1g/L. Red curve measured right after the sonication - Green curve measured after 24 hours.

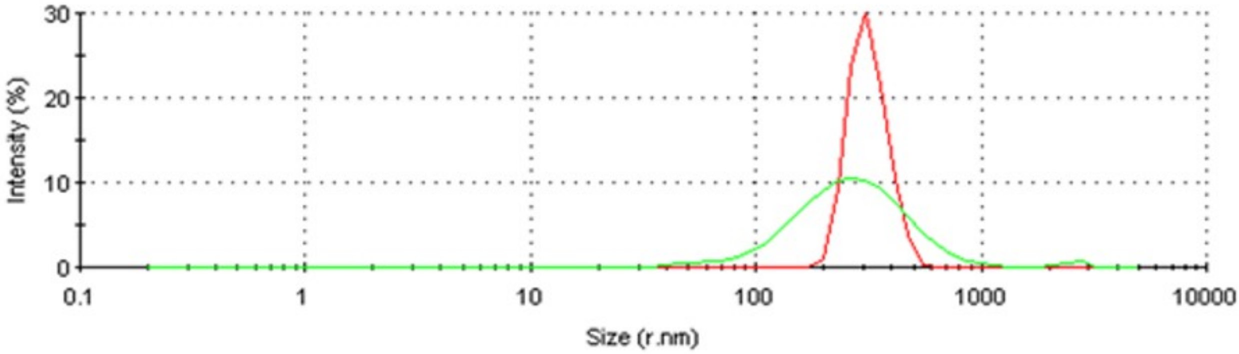

Figure S10. Size distribution by intensity from DLS analysis on the water dispersion prepared using a concentration of precipitate of 0.5g/L. Red curve measured right after the sonication - Green curve measured after 24 hours

**Element Symbol**	**Element Name**	**Atomic Conc. (%)**
C	Carbon	4.2
O	Oxygen	59.9
Na	Sodium	0.2
Si	Silicon	13.9
K	Potassium	2.7
Ca	Calcium	19.0

Table S3. Chemical composition of P11b sample as determined by Electron Dispersive X-Ray (EDX) Analysis expressed as atomic % (average error estimated ± 0.3%).

	Compression strength at different ageing				
Mixture	16 h (N/mm^2)	1 d (N/mm^2)	2 d (N/mm^2)	3 d (N/mm^2)	7 d (N/mm^2)
CEM	8.93 ± 0.10	17.28 ± 0.01	30.33 ± 0.36	36.88 ± 0.28	44.76
CEM-P_{ap}	9.63 ± 0.07	17.8 ± 0.1	30.15 ± 0.42	36.42 ± 0.97	40.47
CEM- P_{350}	10.61 ± 0.29	19.49 ± 0.04	32.36 ± 0.56	39.64 ± 0.14	44.28
CEM- P_{650}	9.49 ± 0.11	17.18 ± 0.14	31.41 ± 1.32	36.39 ± 0.10	44.16

Table S4. Compressive strength of cement mortars samples at different ageing times.

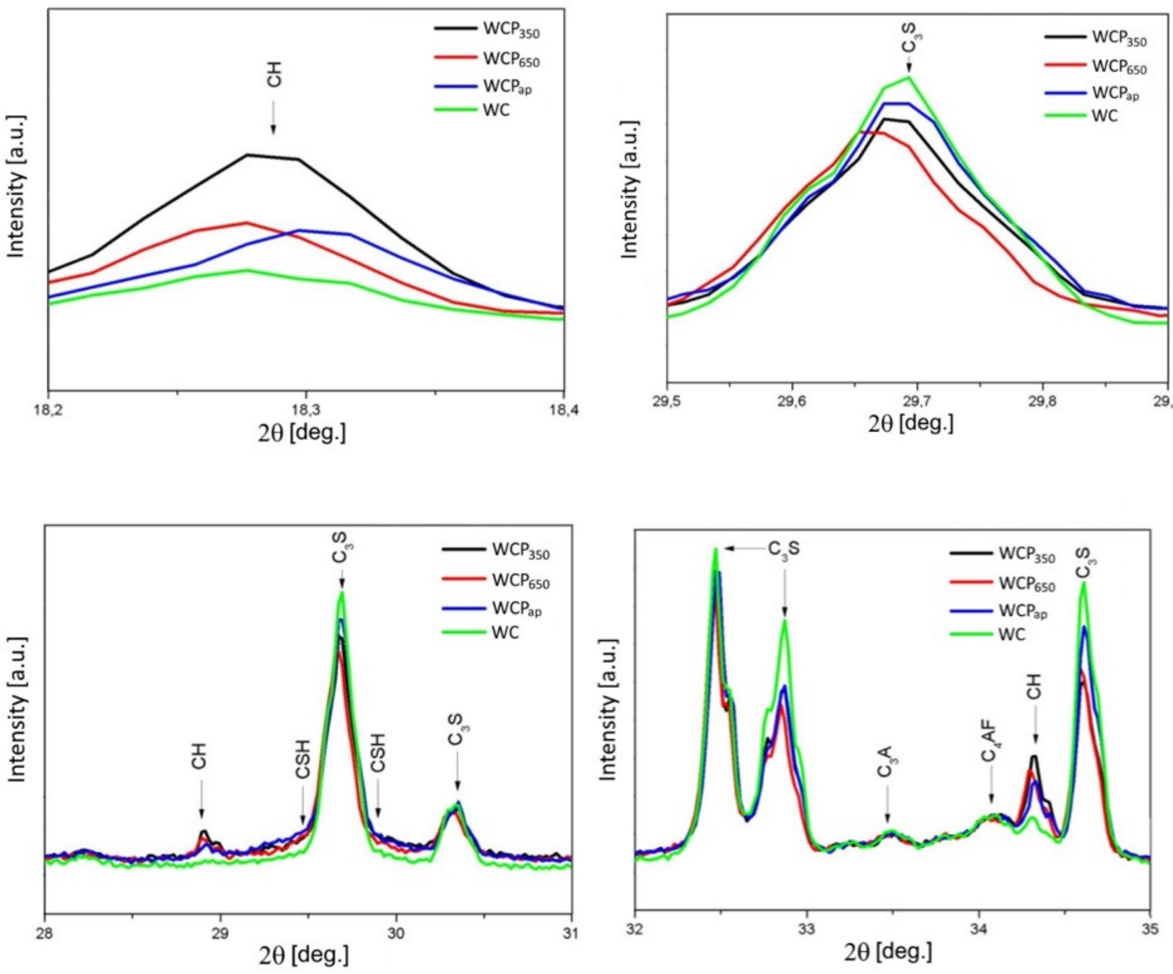

Figure S11. XRPD pattern of the mixtures of additive and cement after hydration.

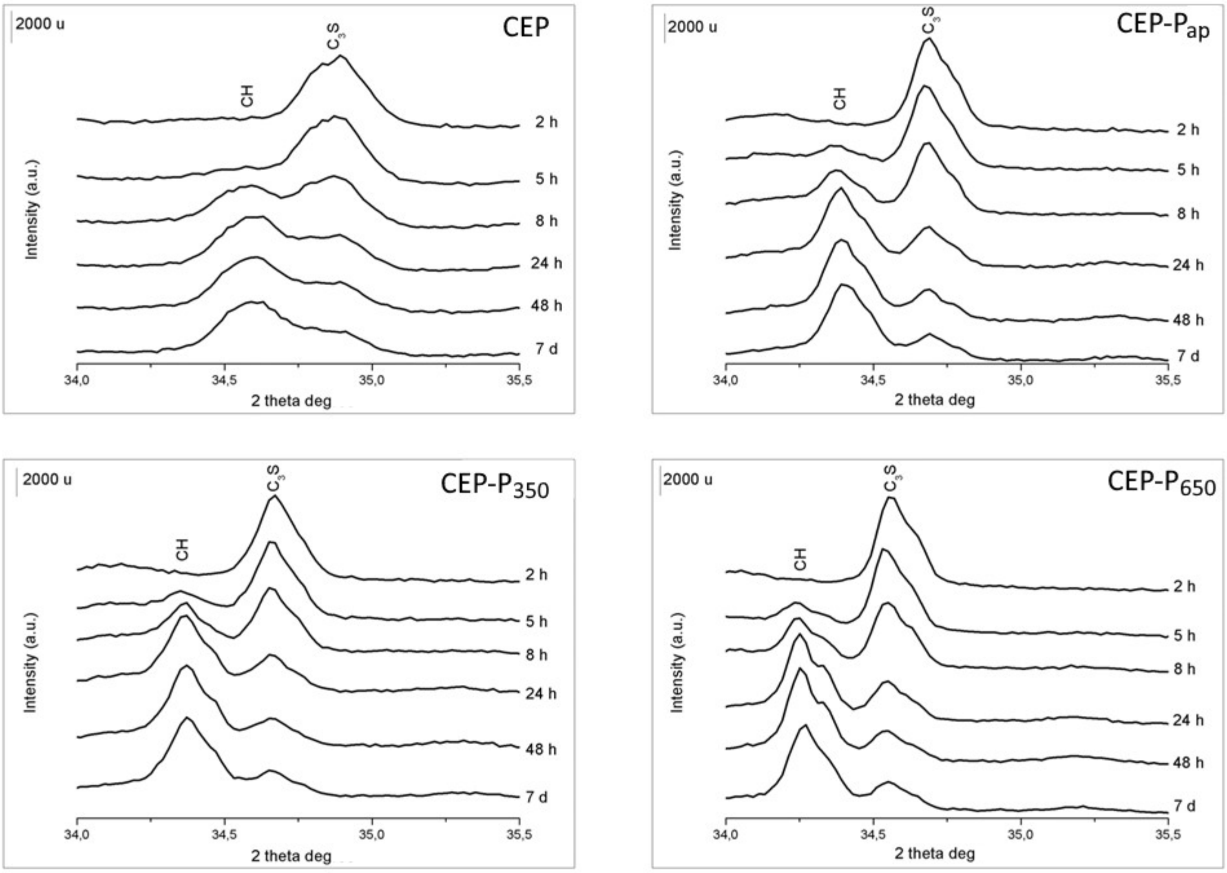

Figure S12 XRPD patterns of the cement paste samples at different hydration times.
